# Temporal control of RNAi reveals both robust and labile feedback loops in the segmentation clock of the red flour beetle

**DOI:** 10.1073/pnas.2318229121

**Published:** 2024-06-12

**Authors:** Felix Kaufholz, Julia Ulrich, Muhammad Salim Hakeemi, Gregor Bucher

**Affiliations:** ^a^Göttingen Graduate School for Neurosciences, Biophysics, and Molecular Biosciences, Göttingen 37077, Germany; ^b^Department of Evolutionary Developmental Genetics, University of Göttingen, Johann-Friedrich-Blumenbach Institute, Göttingen Center for Molecular Biosciences, Göttingen 37077, Germany

**Keywords:** insect segmentation, clock and wavefront, segmentation breakdown, transgenic tool, RNAi

## Abstract

Animals from all major clades have evolved a segmented trunk, reflected in the human spine or the insect segments. These units emerge during embryogenesis from a posterior segment addition zone (SAZ), where repetitive gene activity is regulated by a mechanism described by the clock and wavefront/speed gradient model. In the red flour beetle *Tribolium castaneum*, RNA interference (RNAi) has been used to continuously knock down the function of primary pair-rule genes (pPRGs), caudal or Wnt pathway components, which has led to the complete breakdown of segmentation. However, it has remained untested, if this breakdown was reversible by bringing the missing gene function back to the system. To fill this gap, we established a transgenic system in *T. castaneum*, which allows blocking an ongoing RNAi effect with temporal control by expressing a viral inhibitor of RNAi via heat shock. We show that the *T. castaneum* segmentation machinery was able to reestablish after RNAi targeting the pPRGs *Tc-eve, Tc-odd,* and *Tc-runt* was blocked. However, we observed no rescue after blocking RNAi targeting Wnt pathway components. We conclude that the insect segmentation system contains both robust feedback loops that can reestablish and labile feedback loops that break down irreversibly. This combination may reconcile conflicting needs of the system: Labile systems controlling initiation and maintenance of the SAZ ensure that only one SAZ is formed. Robust feedback loops confer developmental robustness toward external disturbances.

## Significance

The generation of repetitive body parts such as ribs and vertebrae or insect segments has been a very successful evolutionary invention. How these repetitive units form during embryonic segmentation has been of key interest to developmental biologists. So far, they mostly used permanent knock-down of gene function for respective studies in insects. Using a unique tool to end a gene knock-down effect with temporal control, we find both robust and labile feedback loops within the insect segmentation machinery. The robust downstream loops probably buffer ongoing segmentation against external disturbance while the labile upstream loops may ensure that only one trunk is formed.

A striking feature of many animal body plans is their subdivision into repetitive units. Clades with segmented bodies are found in all major branches of animal phylogeny, namely vertebrates, annelids and arthopods (1–3). The repetitive design facilitated the evolution of an amazing morphological and functional diversification along the body axis, contributing to the evolutionary success of these clades. In most vertebrates and arthropods, embryonic segmentation is generated by a posterior clock-like mechanism that uses temporal oscillations of gene activity to generate repetitive spatial patterns (1, 4). Most experiments studying the clock in insects used parental RNAi (pRNAi) (5), which leads to knock-down of gene function from the beginning of development. Hence, these experiments revealed only the first essential function of the respective genes, and later aspects of the segmentation clocks have remained inaccessible to functional investigations. Here, we present a unique tool for shutting down an ongoing RNAi response with temporal control. We use this method to ask the question whether the segmentation clock can reestablish itself after it has broken down as a consequence of a knock-down of key segmentation genes.

**Fig. 1.**
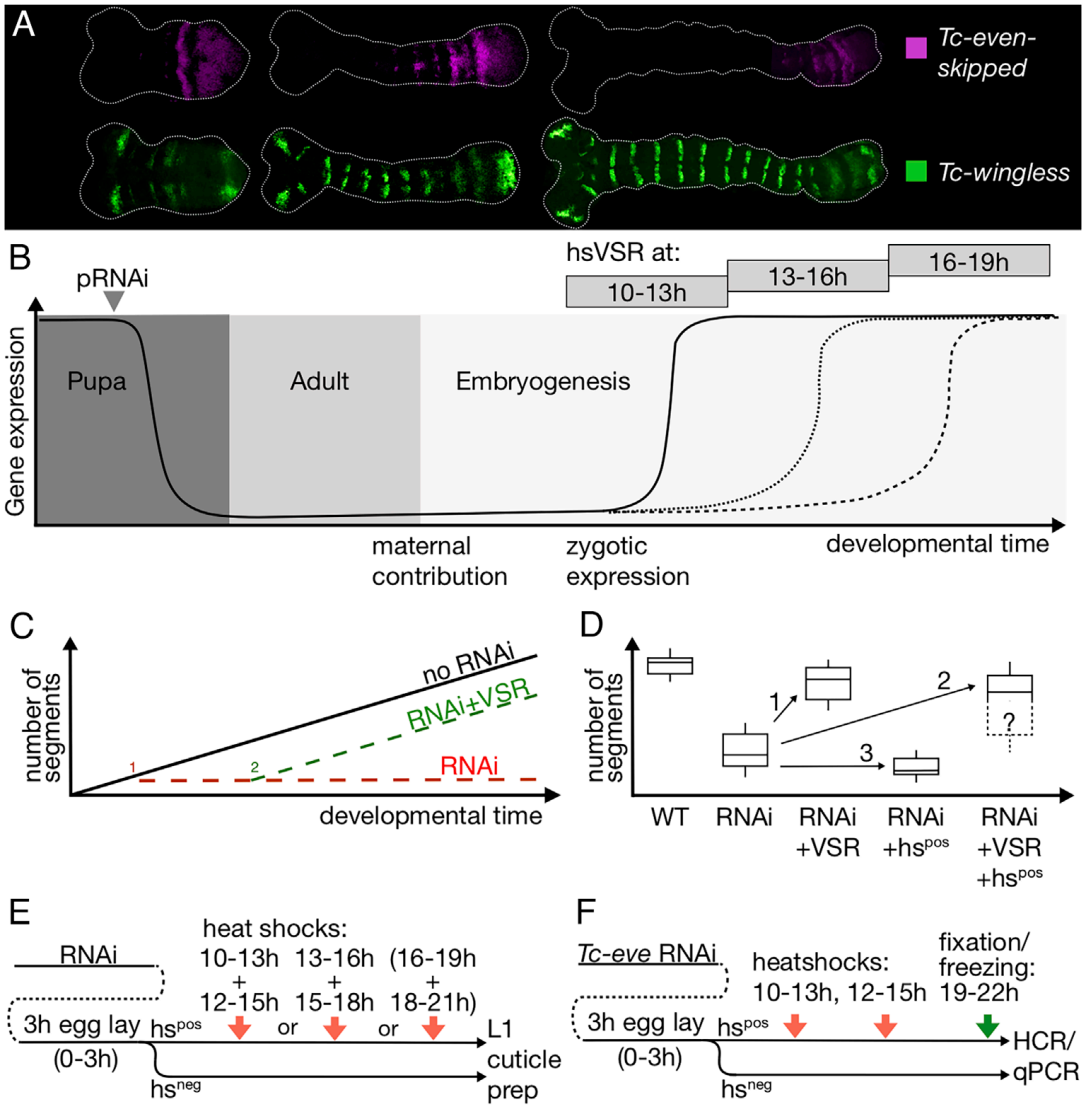
Overview of the experimental design. (*A*) *T. castaneum* embryos representing the stages used for blocking the RNAi (11.5, 14.5, and 17.5 h of development at 32 °C). Shown is the expression of *Tc-eve* as an example for a pPRG and *Tc-wg* as marker for segment boundaries. (*B*) Design of the rescue experiments. After pRNAi, the level of expression drops (declining black line) and is low in the adult and at the beginning of embryogenesis of the offspring. Heat shock–mediated expression of the VSR blocks the RNAi effect after 10 to 13, 13 to 16, or 16 to 19 h after egg laying, respectively. This allows gene expression to resume at different developmental stages (increasing black and dotted lines). (*C*) Blocking the studied genes from the beginning of segmentation blocks the formation of any segments (red broken line). In case of heat shock–mediated rescue (timing: 2), segmentation resumes and forms some posterior segments (green broken line). Anterior segments should not be rescued. (*D*) Several additive effects influence the final phenotype. RNAi lowers the number of segments due to the knock-down of an essential patterning gene. Rescue by the VSR increases the number of segments (arrow 1). However, heat shock as such has negative influence on segmentation making phenotypes stronger (arrow 3). Hence, the final phenotype is a combination of rescue and heat shock defects (arrow 2). (*E*) Details of the procedure. After pRNAi, eggs were collected for 3 h (0 to 3) and treated with heat shocks at different times of development in separate experiments: early (10 to 13 h), intermediate (13 to 16 h), and late (16 to 19 h). To maintain a high level of VSR expression, the heat shock was repeated after 2 h, respectively. The latest heat shock showed minor effects and was not included in all experiments. Hs-negative (hs^neg^) siblings were used as controls. (*F*) For staining the embryos and for the qPCR experiments, the treated embryos were fixed at a stage corresponding to 19 to 22 h of development.

In insects, the process of embryonic segmentation has been best studied in *Drosophila melanogaster melanogaster*, where a hierarchically organized gene regulatory network (GRN) leads to an almost simultaneous formation of all segments (6, 7). In most insects, however, segments are added sequentially from a posterior segment addition zone (SAZ) (1–3). In those animals, a clock-like mechanism seems to sequentially generate the segment boundaries (8–11). Intriguingly, the logic underlying the insect segmentation clock is similar to the one of the vertebrate somitogenesis clock, although the involved genes differ (4, 12, 13). The red flour beetle *T. castaneum* has been the main insect model organism for studying the segmentation clock of insects. Current models on its molecular setup have been discussed in recent reviews (1, 4). In principle, the clock acting in the SAZ consists of two components: A gene or GRN oscillates in a cell-autonomous way in the SAZ, and a posterior to anterior signaling gradient regulates the timing and termination of this oscillation. In the classic clock and wavefront model, the posterior signal forms a retracting front or a steep gradient that determines the boundary where the oscillation is frozen in an elongating tissue. In nonelongating tissues, static gradients govern the speed of the oscillations leading to apparent waves of expression. A recently suggested modification combines these two models by assuming a posterior gradient, that retracts along an elongating tissue and that governs the speed of the oscillations and their freezing. We use the terminology of that model where the posterior gradient is called “speed regulation gradient” (14). This gradient across the SAZ remains stable throughout segmentation and activates the cellular oscillator in a concentration-dependent way. The combination of both components leads to dynamic on and off states of oscillator gene expression in pseudowaves initiating in broad domains at the posterior, moving toward the anterior SAZ while becoming narrower and eventually stalling at the anterior boundary of the SAZ to form a new segment boundary. In this work, we use the concept of “speed regulation” instead of the initially suggested “wavefront” concept, which actually represents an extreme case of speed regulation (15). In *T. castaneum*, Wnt signaling at the posterior pole of the SAZ is autoregulatory, and it activates *Tc-caudal* expression (16–18). A feedback loop between *Tc-caudal* and Wnt signaling has been suggested based on respective spider data and based on the fact that knock-down of both is required for the generation of double-head embryos in *T. castaneum* (16, 19). One or both components may function as the molecular realization of the speed regulation gradient (14, 20). *Tc-caudal*, *Tc-dichaete,* and *Tc-odd-paired* have been termed timing factors reflecting their subsequent functions in *D. melanogaster* segmentation and their expression in the *T. castaneum* in SAZ in patterns compatible with similar temporal input to the clock (21). The primary pair-rule genes (pPRGs) *Tc-even-skipped* (*Tc-eve*), *Tc-runt* (*Tc-run*), and *Tc-odd-skipped* (*Tc-odd*) are the oscillating genes (8, 11) while *Tc-hairy* may have lost an ancestrally essential function in *T. castaneum* (1, 22). Regulatory interactions among the pPRGs are thought to realize the negative feedback loop required for the oscillator. Together, they regulate the expression of the secondary pair-rule genes *Tc-paired* (*Tc-prd*) and *Tc-sloppy-paired*, which eventually turn on the segment polarity genes such as *Tc-wingless* (*Tc-wg*) (23, 24). The expression of *Tc-ev*e in stripes in the SAZ and *Tc-wg* marking established segment boundaries is shown in Fig. 1*A*. Different regulatory interactions among the pPRG oscillator genes have been proposed to explain their expression patterns (8). Different from vertebrates, there is another clock ticking in parallel to the periodic pPRG-based clock. This nonperiodic clock is formed by the *T. castaneum* gap genes and is probably under the control of the same speed regulation gradient. It leads to the one-time sequential activation of the different gap genes (14, 25). While it has become clear that gap genes regulate Hox gene expression for regionalization of the body, they may provide additional input for pPRG regulation as well. Interestingly, the knock-down of several gap gene orthologs led to a complete breakdown of segmentation (22, 26–28) while heat shock–mediated misexpression of *Tc-hunchback* led to a reinitiation of the gap gene cascade (25).

These insights were deduced from modeling, gene expression patterns, and from knocking down gene function by pRNAi. In the latter experimental approach, dsRNA is injected into female beetles, who transmit the RNAi effect to their offspring, which consequently suffers from the RNAi knock-down from the earliest embryonic stages onward. Hence, strictly spoken, the interactions found in these studies are valid for the first rounds of oscillation while later interactions might differ. Unfortunately, the available techniques did not allow studying the later stages of the clock independently from its initiation. As consequence, it has remained unclear, whether the observed breakdown of segmentation after early knock-down of pPRGs was irreversible. As an alternative, a continued depletion of gene function could be required to induce that drastic phenotype. Similarly, it has remained unclear whether the observed Wnt autoregulation at the posterior pole was sufficient for maintaining Wnt ligand expression or, alternatively, whether an as yet unknown upstream factor was required for maintaining Wnt signaling at the posterior pole. Finally, it has remained unclear in how far the oscillating segmentation network active in the SAZ would be able to reestablish itself after it had broken down. Interestingly, the nonoscillatory gap-gene cascade could indeed be reinitiated by heat shock–mediated misexpression of the first gene of the cascade, namely *Tc-hunchback* (25).

In order to answer these questions and to open up additional experimental possibilities more generally, we developed a system for blocking an ongoing RNAi response with temporal control. For that purpose, we used viral suppressors of RNAi (VSRs), which are proteins that evolved to rescue viruses from the RNAi immune response of the host. We found that heat shock–mediated expression of one VSR, CrPV1A, efficiently blocked the RNAi response in *T. castaneum*. Using this tool, we found that the segmentation breakdown due to pPRG RNAi was reversible, i.e., the system reestablished itself once the knock-down was suppressed. In contrast, the breakdown observed after knocking down Wnt signaling components was irreversible. This is evidence that the Wnt autoregulatory loop is at the top of speed regulation gradient maintenance.

## Results

### Establishing a Viral Suppressor of RNAi as a Tool in *T. castaneum*.

RNAi is an antiviral defense and viruses evolved proteins to interfere with that process. A variety of VSRs from insect and plant viruses have been described (29–36). Based on the conservation of the proteins involved in RNAi (37) and the proven functionality of some of these inhibitors in flies, we assumed that VSRs might be able to block RNAi in *T. castaneum* as well. To test this, we generated transgenic lines for six VSRs where the VSR expression was under the control of the Gal4-controlled UAS enhancer (38, 39). These lines were tested for their efficacy in suppressing RNAi in *T. castaneum* by two independent tests. We used two different Gal4 driver lines and tested the rescue of an endogenous gene and a heterologous gene expressed from a transgenic construct (see *SI Appendix, Text 1*, for experimental details and results). Only the VSR CrPV1A derived from the *Cricket Paralysis Virus* showed strong reduction of the RNAi effect in both tests while FHV B2 from the *Flock House Virus* showed some effect in one test (see *SI Appendix, Text 1*). Hence, we decided to use CrPV1A for our purpose. The CrPV1A protein is responsible for the high pathogenicity of the *Cricket Paralysis Virus* by interacting with the endonuclease Ago-2, a component of the RISC complex (see *SI Appendix, Text 2* for further information). In *D. melanogaster*, it did not interfere with the miRNA pathway (33). However, we were not able to generate transgenic lines with a high level of ubiquitous CrPV1A activity despite many trials. Therefore, we hypothesize that strong ubiquitous VSR expression may affect viability—possibly via blocking the miRNA pathway. Taken together, our results identified CrPV1A as a potent inhibitor of RNAi in *T. castaneum*.

### Temporal Control of RNAi by Heat Shock–Mediated VSR Expression.

**Fig. 2.**
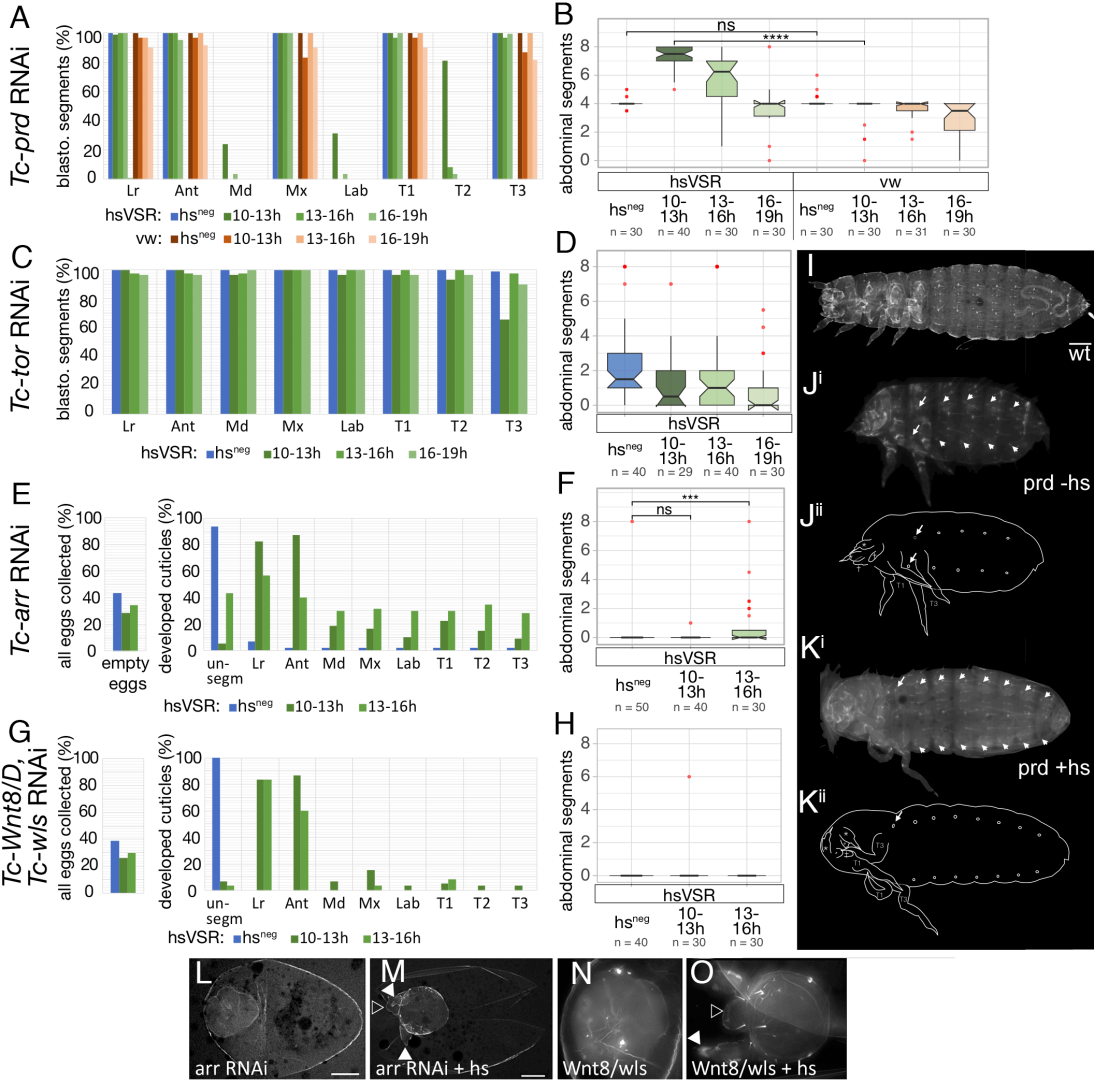
Testing the hsVSR system and rescuing the Wnt pathway component. (*A*) *Tc-paired* was used as positive control. The formation of anterior segments, which are built from the blastodermal fate map, was scored after pRNAi in the hsVSR line and heat shock treatments after different times after egg laying (green bars). As negative controls, knock-down embryos of the hsVSR line without heat shock were analyzed (hs^neg^, blue bars) as well as wild-type animals (vermillion^white^ strain, *vw*) with RNAi and heat shock (brownish bars). The presence of respective structures was quantified (Lr: labrum; Ant: antenna; Md: mandible; Mx: maxilla; Lab: labium; T1-3: thoracic segments). As expected, Md, Lab, and T2 were mostly absent after RNAi. Only the early heat shock treatment in the hsVSR line (10 to 13 h, dark green bars) showed clear rescue. In line with segmentation proceeding from anterior to posterior, the posterior segments were rescued more frequently. (*B*) In the same animals, the number of abdominal segments was counted. In the wt RNAi control (right part, brownish boxes), the number of segments was reduced to four by RNAi as expected (eight abdominal segments in the wild type). The same was found for the non-heat shock control of the hsVSR line (leftmost box, hs^neg^). Rescue was observed in the heat-shocked hsVSR animals. It was strongest for the earliest heat shock (dark green box) while no rescue was observed in the latest (light green box). As an unspecific side effect, the heat shock treatment increased the severity of the RNAi treatment in both hsVSR and wt animals (reduced number of segments seen in right-most green and brownish boxes). (*C* and *D*) The same treatments were performed for the negative control *Tc-tor*. No rescue was observed as expected for this gene, which is active in the SAZ only at the initiation of segmentation. (*E*–*H*) Knock-down of Wnt components is known to lead to empty-egg phenotypes, where embryogenesis stops before secretion of a cuticle. Therefore, we documented the portion of empty-egg phenotypes in all collected embryos (*Left* in *E* and *G*) and analyzed the subset of embryos with cuticle both for the anterior morphological structures (*Right* in *E*) and the number of abdominal segments (*F*). We found no rescue for Wnt8/wsl double RNAi (*G* and *H*). Some rescue of anterior structures and abdominal segments is observed for *Tc-arr* (*E*). However, we assign this effect to later functions of Wnt, which are independent of its SAZ function (see text for arguments). (*I* and *J*) In *Tc-prd* RNAi cuticles, loss of anterior and abdominal segments is observed (compare *Ji* with *I*). (*K*) After heat shock–mediated rescue, the anterior defects are still observed (white arrows) while the posterior abdominal segments are rescued (white arrowheads mark segmental tracheal openings). (*L* and *M*) *Tc-arr* RNAi leads to spherical cuticles that lack distinguishable structures. After hsVSR rescue, antennae (white arrowheads) and labrum (open arrowhead) are rescued but posterior segments are not. The outer cuticle sphere is the extraembryonic vitelline membrane. Scale as in *I*–*K* to visualize the size difference or resulting cuticles. (*N* and *O*) After *Tc-Wnt8/D-Tc-wls* double RNAi, spherical cuticles without external structures develop. Upon hsVSR treatment, antennae and labrum are rescued. Scale increased to visualize details—the actual size of the cuticles is comparable to *L* and *M*.

In order to gain temporal control on RNAi, we established transgenic lines where CrPV1A was under the control of the *T. castaneum* heat shock promoter (hsVSR) (40). In order to test the hsVSR for applicability for the segmentation process, we optimized the procedure and performed several control experiments. As positive control, we chose the secondary pair-rule gene *Tc-paired (Tc-prd),* which is a downstream gene of the segmentation clock (23, 24). Therefore, rescue of segmentation by VSR expression was expected because the segmentation clock does not breakdown in *Tc-prd* RNAi. We performed pRNAi of *Tc-prd* in our hsVSR line. The RNAi embryos were either not heat shocked or were treated with heat shocks during one of three different time windows during germ band elongation (see the scheme in Fig. 1*B*). In the absence of a heat shock, the L1 larval cuticles displayed the published pair-rule-gene phenotype where the number of abdominal segments was halved to a median of four abdominal segments (Fig. 2 *A* and *B*, hs^neg^, Fig. 2*J*) (23, 24). Heat shock–mediated VSR expression at 10 to 13 h after egg laying (32 °C) rescued the abdominal segment number to a median of 7.5 abdominal segments, i.e., almost to the wild type (Fig. 2*B*, hsVSR 10 to 13 h, Fig. 2*K*). Some rescue of more anterior segments was observed as well, i.e., Md (25%), Lab (30%), and the second thoracic segment (80%) (Fig. 2*A*). Later VSR expression (13 to 16 h after egg laying) rescued to a median number of only six abdominal segments (Fig. 2*B*, hsVSR 13 to 16 h) while the latest heat shock (16 to 19 h) failed to rescue abdominal segments (Fig. 2*B*, hsVSR 16 to 19 h). In our negative controls, i.e., *Tc-prd* RNAi in the *vermillion white* (*vw*) wild type, the cuticles showed no rescue irrespective of whether heat shock was applied or not (Fig. 2 *A* and *B*). Taken together, this experiment showed that rescue of the segmentation process from an ongoing RNAi effect was possible by blocking RNAi with our hsVSR (see *SI Appendix*, Fig. S1 *G* and *H* for an independent replicate of this experiment done by another experimenter).

As negative control, we tested *Tc-torso* (*Tc-tor*). In *T. castaneum*, Torso signaling is active at the posterior pole in early embryos but not during elongation. Hence, it was suggested to be required for establishment of the SAZ and to initiate posterior elongation but was unlikely to be required for maintaining it (41). Therefore, blocking RNAi targeting Torso signaling during elongation should not have an effect on posterior segmentation. In line with previous results, our RNAi experiments targeting of *Tc-tor* resulted in the loss of most abdominal segments. A median of 1.5 abdominal segments remained (blue box in Fig. 2*D*) while the anterior segments remained unaffected (Fig. 2*C*) (41). As predicted, no rescue of abdominal segments was observed by hs-induced VSR expression for neither time point (green boxes in Fig. 2*D*).

To test for unspecific effects induced by the hs treatment, we performed the same experiments in the hsVSR line and the vw wild-type strain, which is the genetic background for the hsVSR line. Indeed, the heat shock treatment alone led to some reduction of abdominal segments. This was observed in heat-shocked animals of wild type (Fig. 2*B*, light red boxes) and in hsVSR animals without previous RNAi treatment (*SI Appendix*, Fig. S1*F*). In line with this apparently nonspecific effect of heat shocks and/or VSR expression, the RNAi defects increased in heat-shocked animals in both *Tc-tor* (Fig. 2*D*—compare green to blue boxes) and *Tc-paired* knock-down embryos (*SI Appendix*, Fig. S1*H*). We also compared the expression of *Tc-cad*, *Tc-eve,* and *Tc-wg* in wild-type animals with and without heat shock. Besides many embryos with patterns indistinguishable from the wild type, we found an increase of aberrant patterns for *Tc-wg* in the heat-shocked animals, reflecting our observation at the cuticle level (*SI Appendix, Text 3*). Together with previous results by others, who found no change in gap gene expression in heat-shocked embryos (25), we conclude that a heat shock alone leads to a limited increase of unspecific patterning defects that was unlikely to interfere with our approach. In conclusion, the overall effects observed after RNAi and heat shock–induced rescue are composed of three additive effects: first, the reduction of segments resulting from the specific RNAi effect (Fig. 1*D*—second box); second, additional reduction due to unspecific heat shock and/or VSR defects (Fig. 1*D* arrow 3); and third, rescue by blocking the RNAi effect by VSR expression (Fig. 1*D*, arrow 1). The observed overall phenotype results from the combination of these partially opposing effects (Fig. 1*D* arrow 2).

In summary, these proof-of-principle experiments showed that the hsVSR system was able to inhibit an ongoing RNAi response where the 10 to 13 h time window (and to lesser degree the 13 to 16 h window) appeared optimal for effects on the segmentation process. Further, they revealed side effects induced by the heat shock treatment.

### Interfering with Wnt Signaling Leads to an Irreversible Segmentation Breakdown.

pRNAi targeting several segmentation genes led to the loss of all posterior segments, indicating a breakdown of the segmentation machinery. This phenotype is observed for some gap gene orthologs, pPRGs, the terminal gene torso and two components of the SAZ, namely caudal and Wnt signaling (8, 26–28, 41–43). In all these experiments, the genes were knocked down throughout development by pRNAi. Therefore, it has remained unclear whether the phenotype reflected an irreversible breakdown of segmentation or whether continued depletion of the respective component was required for the continuous loss of posterior segments.

Our system allowed us to ask whether the segmentation breakdown observed in those RNAi experiments was reversible or not. Wnt signaling and *Tc-caudal* expression are found in the SAZ throughout elongation and respective RNAi experiments led to a segmentation breakdown (42, 43). It was suggested that Wnt regulates *Tc-caudal*, which in turn represents a speed regulation gradient, which is required to regulate the segmentation clock acting in the SAZ. (4, 20). Indeed, autoregulation of Wnt signaling and activation of *Tc-caudal* by Wnt signaling was shown previously for *T. castaneum* (18, 20). It should be noted that in more basal insects and a spider, interfering with Wnt signaling had similar drastic effects on segmentation, but the Wnt-cad interactions suggested above were not fully confirmed there (44). At least in *T. castaneum*, an autoregulatory loop is suggested to ensure the continuous expression of these components in the SAZ. Hence, interrupting the loop could lead to an irreversible breakdown. Alternatively, if a so far unknown upstream signal located in the posterior SAZ was required for their maintenance, the system would be able to reestablish itself. In order to distinguish between these possibilities, we analyzed *Tc-WntD/8,* which together with the Wnt pathway component *Tc-wntless* (*Tc-wls*) is required for posterior segmentation, which is also true for the Wnt receptor *Tc-arrow* (*Tc-arr*) (42, 45). Besides that role in the SAZ, Wnt is also required for later aspects of segmentation such as the formation of parasegment boundaries. Therefore, the RNAi phenotypes are a mix of early and late functions, which needs to be considered when interpreting the rescue effect. In line with published results, our *Tc-WntD/8+Tc-wls* double RNAi and *Tc-arrow* single RNAi resulted in two classes of phenotypes: completely unsegmented cuticles and “empty egg phenotypes” (ee-phenotype). The ee-phenotype describes eggshells that do not contain embryonic cuticle, because the embryos stopped development before secreting cuticle. The former are a combination of early and late segmentation defects. We found roughly 40 to 45% empty eggs for both RNAi treatments (Fig. 2 *E* and *G*, blue bar in *Left*). Most cuticles showed an unsegmented phenotype (90 to 100%) (blue bars in the “unsegm” column of the right panel in Fig. 2 *E* and *G*; see Fig. 2 *L* and *N* for exemplary cuticles). hsVSR expression at 10 to 13 h slightly reduced the portion of the ee-phenotype (Fig. 2 *E* and *G*, green bars in *Left*). Strikingly, the portion of unsegmented cuticles decreased dramatically (to roughly 5% for both RNAi treatments, see Fig. 2 *E* and *G*, compare green to blue bars). The anterior pre-gnathal structures (labrum and antennae) were rescued more strongly than gnathal and thoracic segments (Fig. 2 *M* and *O*). However, no rescue of abdominal segments was observed for the early treatment (Fig. 2 *F* and *H*; 10 to 13 h). Later VSR expression (13 to 16 h) showed a similar result (Fig. 2 *E* and *G*; light green bars), but we observed some cuticles with an increased number of abdominal segments after late VSR treatment in the *Tc-arr* RNAi but not *Tc-WntD/8;Tc-wls* RNAi (Fig. 2*F*, 13 to 16 h). The lack of posterior rescue cannot be due to failed RNAi inhibition because the clear anterior rescue shows effective inhibition of RNAi by our hsVSR (see *SI Appendix*, Fig. S2 for a biological replicate of both experiments done by another experimenter with similar results). We ascribe the minor posterior rescue seen in the *Tc-arr* experiments (Fig. 2 *E* and *F*) to the mentioned later Wnt functions (e.g., the formation of segment boundaries) for two reasons: First, the rescue effect increased with the later heat shocks. This is in contrast to the expectation for upstream components of the SAZ, where a later rescue should only be able to rescue the most posterior segments as was observed in our positive control *Tc-prd* (Fig. 2 *A* and *B*). Second, those structures, which form independently of the SAZ but need other aspects of Wnt signaling (labrum and antenna), are rescued to a high degree. Third, we do not see rescue when targeting Wnt8, which is exclusively expressed in the SAZ (46).

In summary, our analyses indicated that the breakdown of abdominal segmentation after loss of Wnt signaling was irreversible, indicating the interruption of an essential autoregulatory loop of Wnt signaling alone or autoregulatory interactions between Wnt-caudal. However, we cannot rule out minor rescue effects that might have been blurred by the additional negative defects induced by the heat shock. We were not able to test *Tc-caudal* because pRNAi leads to sterility prohibiting the collection of the high number of embryos required for these types of experiments.

### Segmentation Breakdown after pPRGs Knock-Down Is Reversible.

**Fig. 3.**
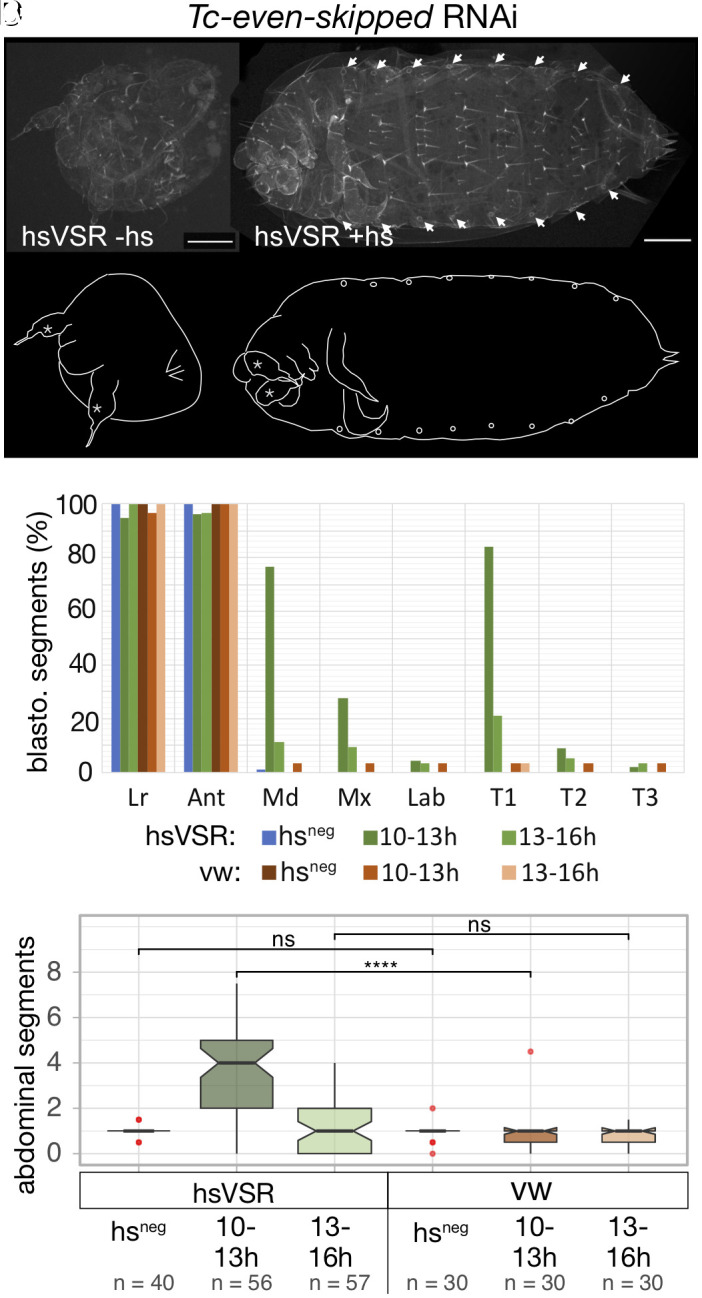
Reestablishment of segmentation after the rescue of *Tc-eve* expression. (*A*) *Tc-eve* RNAi leads to complete loss of trunk segmentation—only the labrum, the antennae, and the terminal urogomphi can still be discerned in the resulting cuticle balls. (*B*) After hsVSR rescue, some anterior segments in addition to some posterior abdominal segments are rescued—in the specimen shown, all eight abdominal segments are discernable. (*C*) Quantification of the effects for the blastodermal segments reveals the highest degree of rescue for the early treatment. (*D*) Likewise, the strongest rescue of abdominal segments is found for the early rescue (dark green; 10 to 13 h). Labeling as in Fig. 2.

Downstream of the Wnt signaling- and *Tc-caudal* gradients, three pPRG are essential for segmentation. *Tc-eve*, *Tc-run*, and *Tc-odd-skipped* (*Tc-odd*) form a regulatory circuit leading to their oscillating expression in the SAZ. The resulting overlapping stripes provide spatial information for segmentation (10, 11; models discussed in refs. 1, 4, 8, and 14). RNAi knock-down of each pPRG leads to the breakdown of blastodermal and posterior segmentation (8). It was suggested that their mutual regulation represented a regulatory circuit, which had to be started at the blastoderm stage and stopped after elongation was completed (8). Later, it was suggested that their oscillations were under the control of a speed regulation gradient provided by ongoing expression of *Tc-caudal* in the SAZ (10, 14, 20). In a scenario with a fully autonomous regulatory circuit, the breakdown would be irreversible while in the model involving a speed regulation gradient, reestablishment of segmentation under the control of the unaffected upstream Wnt/*Tc-caudal* function was likely. As previously shown, *Tc-eve* RNAi resulted in cuticles that retained only labrum (Lr) and antennae (Ant) in both the hsVSR line and the wild-type controls (Fig. 3 *A* and *C*, “hs^neg^”). In contrast to previous results, we noted a pair of tracheal openings (90%) in these RNAi embryos. Expression of the VSR during the early time window (10 to 13 h) did rescue both some anterior and abdominal segments (Fig. 3 *C* and *D*, green bars and boxes). Rescue of Md, Mx (maxillae), and one thoracic segment was observed (probably T1 as judged by the absence of a tracheal opening) (Fig. 3*C*). The median number of abdominal segments increased to four segments (with some cuticles showing as many as 6 to 7 abdominal segments, see Fig. 3*D*). The hs-treated wild-type control did not show any rescue (Fig. 3 *C* and *D*; red bars and boxes). VSR expression at 13 to 16 h led to no significant rescue. However, some cuticles actually had more than the expected number of abdominal segments but this apparent rescue was counterbalanced by cuticles with additional loss of segments (Fig. 3*D*, “hsVSR, 13 to 16”). Likewise, the vw controls showed additional loss of segments upon heat shock. Hence, it is possible that the negative effect of the hs treatment counterbalanced a minor rescue effect at the late time window (Fig. 1*D*). This experiment was repeated two more times, where one experiment showed similar results and one revealed no rescue effects (*SI Appendix*, Fig. S3).

**Fig. 4.**
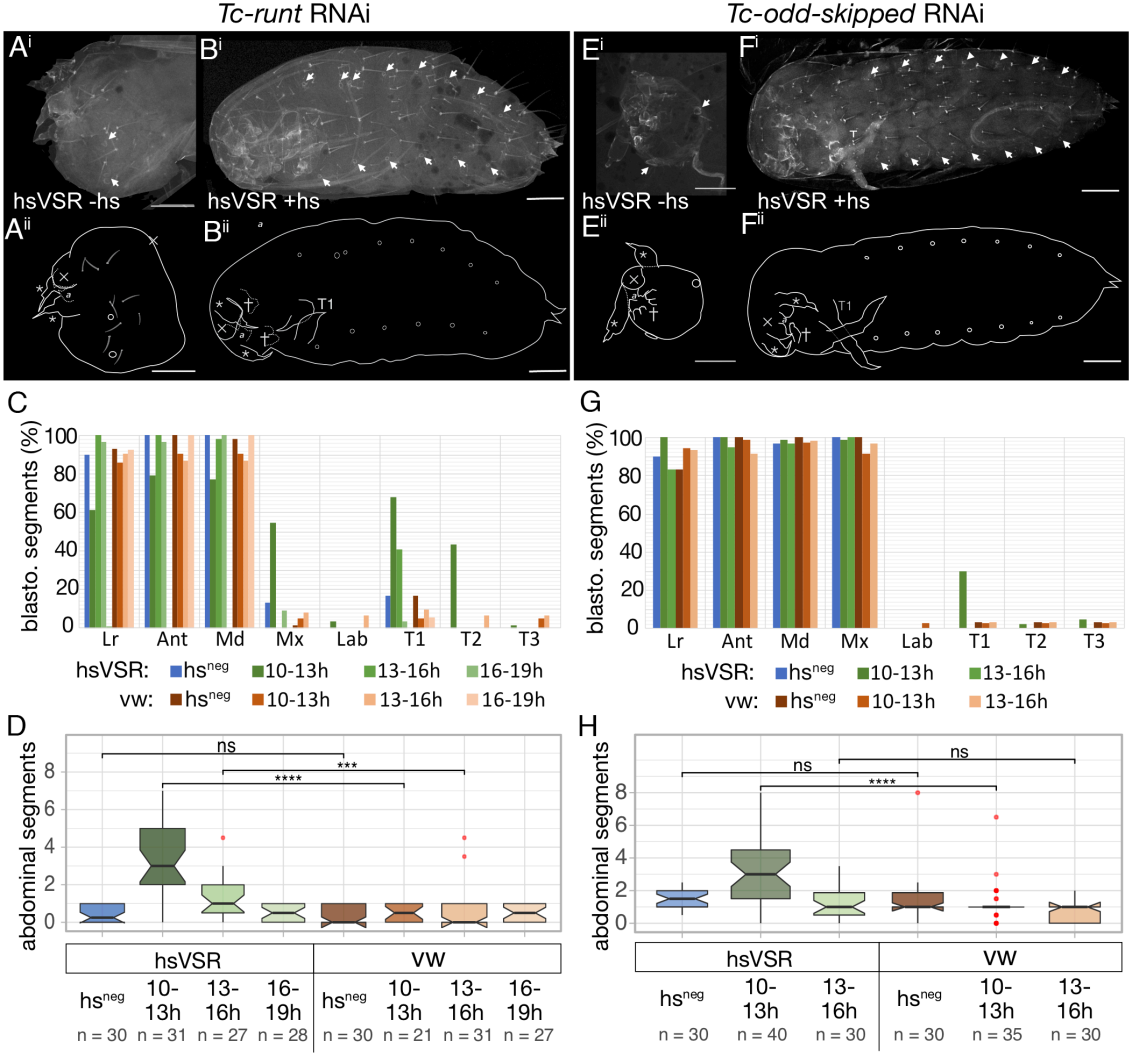
Reestablishment of segmentation after the rescue of *Tc-run* and *Tc-odd* expression. (*A* and *B*) The *Tc-run* RNAi phenotype (*A*) is rescued after the hs treatment (*B*). (*C* and *D*) Quantification of the effects for the blastodermal (*C*) and the abdominal segments (*D*) reveals the highest degree of rescue for the early treatment. (*E* and *F*) Cuticles of *Tc-odd* RNAi phenotypes without (*E*) and with hsVSR rescue (*F*). (*G* and *H*) The rescue of anterior segments (*G*) and abdominal segments (*H*) was quantified. The rescue of blastodermal segments is weaker in *Tc-run* and *Tc-odd* compared to *Tc-eve*. Labeling as in Fig. 2.

As previously published, parental *Tc-run* RNAi resulted in cuticles that carried mandibles and up to one abdominal segment (Fig. 4 *A* and *C*). Early VSR expression (10 to 13 h) rescued some blastodermal segments (mx, T1, T2) and the abdominal segments to a median number of three (Fig. 4 *B*–*D*). Some cuticles showed five or more rescued abdominal segments (Fig. 4*D*). Rescue at 13 to 16 h showed a similar albeit weaker rescue. Two more repetitions by the same researcher revealed no effect while a repetition by another researcher confirmed the rescue (*SI Appendix*, Fig. S4).

*Tc-odd* pRNAi knock-down resulted in cuticles missing all segments posterior to the Mx (Fig. 4*E*), as expected. Only the early window of VSR expression (10 to 13 h) significantly rescued abdominal segments to a median number of three segments (Fig. 4 *F* and *H*). Of note, a small number of cuticles showed up to eight rescued segments. Again, two more repetitions gave unclear results while the repetition by another scientist showed a clear effect (*SI Appendix*, Fig. S4).

In summary, for all three pPRG, we found that segmentation could be reinitiated after a breakdown. Interestingly, the rescue for the pPRGs was mostly restricted to the earliest time window of RNAi suppression while the rescue of the secondary PRG *Tc-prd* was found also for later time windows. Due to the complex setup, the strict timing requirements and likely variability in the heat shocks of these experiments, not all experiments led to rescue. However, for each pPRG, we found rescue of segmentation in at least two independent replicates and the combination of posterior rescue with anterior deletions is a very specific and unique phenotype. Together with the expression analysis presented below, this makes us confident that the results are valid.

### Gene Expression Patterns Reflect Early Rescue by hsVSR Treatment.

Our results on the cuticle level indicated that the segmentation machinery could be reestablished after breakdown. However, cuticle is secreted at the end of embryonic development (which takes roughly 72 h at 32 °C) while segmentation takes place during the first 24 h. Hence, late compensatory effects could blur the early direct effects of the rescue. Therefore, we wanted to observe the reestablishment of the segmentation clock more directly after VSR expression. *Tc-eve* RNAi was chosen for that purpose because it had shown the most robust response in our previous experiments. We repeated the experiment and checked a portion of the embryos for successful rescue on the cuticle level to confirm successful performance of the experiment. The other embryos were fixed some hours after the hsVSR treatment to visualize the expression of the three pPRGs and the segmental marker *Tc-wg* (see Fig. 1*F* for experimental outline). Of note, we had to fix wt and heat-shocked embryos at different times in order to obtain comparable stages because heat shock leads to a delay in development for which we had to compensate. Hence, we first carefully staged *Tc-wg* patterns (*SI Appendix*, Fig. S5) and optimized the timing of fixation such that animals from the different experimental groups (with and without heat shock, with and without *Tc-eve* RNAi) would be at comparable stages (*SI Appendix*, Fig. S6).

**Fig. 5.**
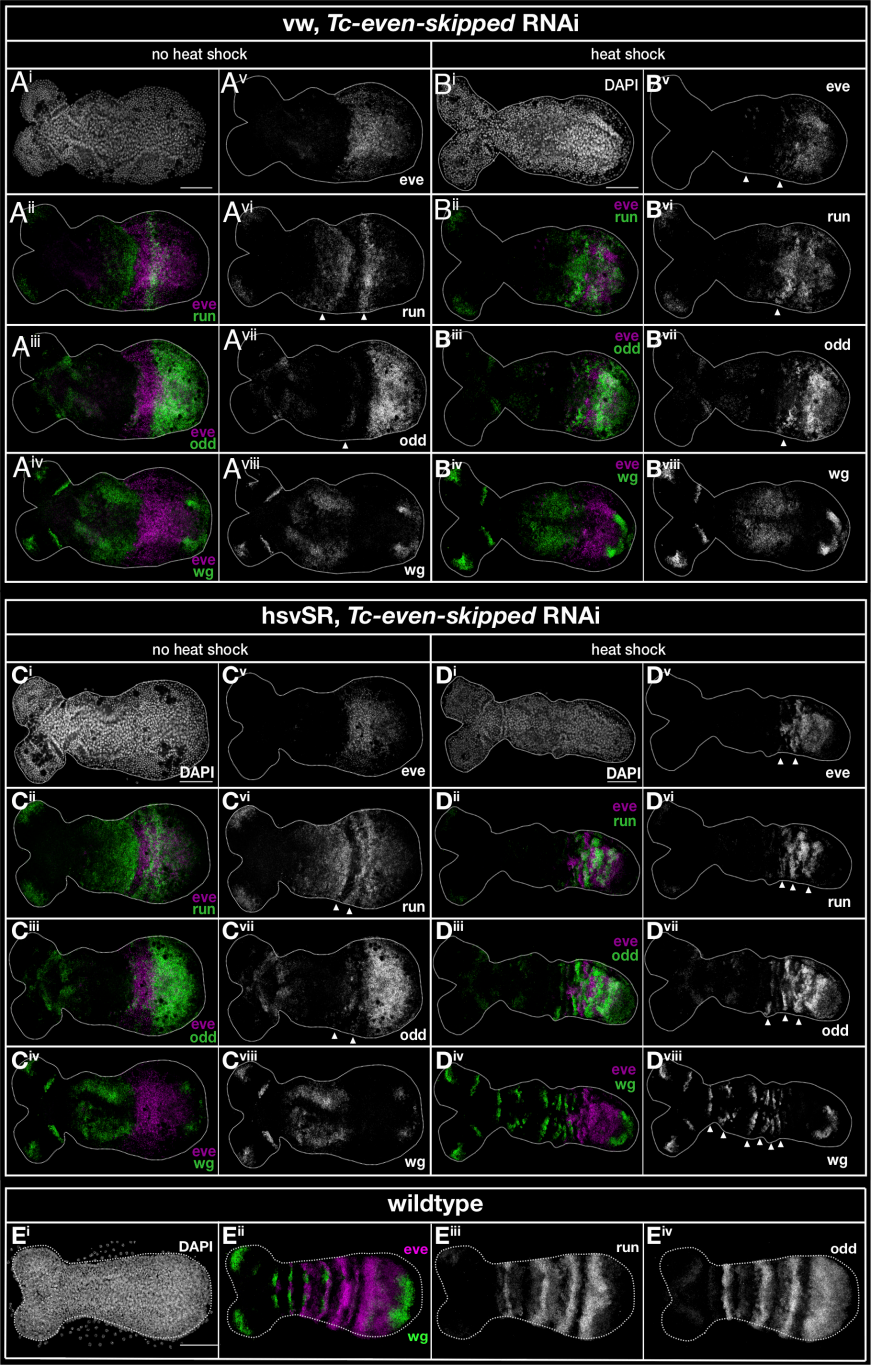
Expression of pPRGs and Tc-wg in Tc-eve RNAi embryos with and without hsVSR rescue. (*A* and *B*) The expression of the pPRGs and *Tc-wg* is severed in wild-type embryos after *Tc-eve* RNAi (*A*). Heat shock alone does not rescue the defects (*B*). The morphology of the embryo is shown by DAPI staining (*Ai* and *Bi*). The quadruple HCR in situ staining in those embryos is shown in combinations of two genes, respectively (*Left* column), and for each gene separately as grayscale picture (*Right* column, respectively). (*C* and *D*) RNAi in the hsVSR line leads to defects comparable to RNAi in the wild type (*C*, compare with *A* or *B*). However, hs treatment induces the emergence of stripes (*D*). (*E*) The expression patterns are shown in the wild type without RNAi treatment.

pRNAi of *Tc-eve* in the wild type resulted in the almost complete loss of segmentation irrespective of heat shock treatment. Instead of segmental stripes, a broad *Tc-wg* domain was observed in the trunk (Fig. 5 *Aiv* and *Biv*). *Tc-eve* formed one broad domain without stripes (Fig. 5 *Aiv* and *Biv*), which did not overlap with the *Tc-wg* pattern. This pattern was not observed in heat-shocked wild-type animals (*SI Appendix, Text 3*). Likewise, *Tc-odd* was expressed in one domain while *Tc-run* showed two abnormal stripes (Fig. 5 *Aii* and *Bii*—compare to wild-type patterns in Fig. 5*E*). *Tc-eve* RNAi in the hs-VSR line without heat shock led to essentially the same patterns (compare Fig. 5*C* to Fig. 5 *A* and *B*). However, upon hsVSR treatment, the expression of *Tc-wg* reflected the formation of stripes (Fig. 5*Div*—compare to Fig. 5 *Aiv*, *Biv*, and *Civ*). In addition, all three pPRGs regained some degree of striped expression (Fig. 5*D*). To quantify this rescue, we counted the number of stripes of the pPRGs and *Tc-wg* in a number of embryos. Indeed, we found a highly significant increase of stripes after rescue for *Tc-wg* while the low number of pPRG stripes visible at the same time (maximum three) led to a low level of statistical significance (compare the values after heat shock in *SI Appendix*, Fig. S7*A*). In an alternative approach, we sorted the stained germband embryos into three classes based on their overall expression patterns: “Close to WT” (WT), “intermediate” (+/−), and “all stripes lost” (−). (*SI Appendix*, Fig. S7*B*; see *SI Appendix*, Fig. S8 for documentation of pictures and our embryo classification). For all three pPRGs, the highest portion of WT and intermediate phenotypes was found in the hsVSR treated batches (*SI Appendix*, Fig. S7*B* hs-VSR, 10 to 13 h). For *Tc-eve*, the difference was statistically significant while for the other genes, the p-value was low but did not reach significance levels (*SI Appendix*, Fig. S7*B*).

### Self-Repressing Function of *Tc-eve* Revealed by qPCR.

Finally, we sought to check for up- and downregulation of the involved genes by qPCR. We confirmed strong increase of VSR expression upon heat shock (*SI Appendix*, Fig. S9). Expression of *Tc-odd* and *Tc-rut* was not much altered in line with our expression analysis, where both genes remain expressed but lose their striped patterns (Fig. 5). Surprisingly, RNAi targeting *Tc-eve* did not reduce the *Tc-eve* transcript level. However, when testing for intronic sequences in *Tc-eve* RNAi embryos, we found a strong upregulation of expression (*SI Appendix*, Fig. S9). Apparently, the loss of *Tc-eve* function leads to upregulation of its expression, blurring the qPCR results. This result is a strong indication for a so far ignored self-repressing function of *Tc-eve* during segmentation. In line with this assumption, *Tc-eve* RNAi embryos that were rescued with hsVSR returned to normal intronic expression levels (*SI Appendix*, Fig. S9).

## Discussion

### Gaining Temporal Control on RNAi by hsVSR—Emerging Possibilities and Restrictions.

With this work, we expand the toolkit of *T. castaneum* with a system that allows to block RNAi with temporal control. The tool might be useful to distinguish between early and late function of genes (shown in this study) or for the analysis of other temporal processes such as the sequential expression of neuroblast timing factors. Further, it could help to overcome technical problems with pRNAi of genes that lead to sterility. To that end, blocking RNAi in the mother but not the offspring could reduce the sterility issue. We note that the heat shock experiments were sensitive to changes in the procedure and had to be optimized carefully. Importantly, we show that the negative effect of a heat shock treatment on developmental processes has to be controlled for. Given the documented function of CrPV1A in flies and beetles, it seems likely that it will be active in other insects, too, opening the possibility to transfer that technique to other species. Theoretically, the CrPV1A VSR could also be used for tools that allow spatial control of RNAi. However, from several unsuccessful attempts in that direction we conclude that strong ubiquitous expression of the CrPV1A probably has negative effects on viability. This may interfere with the establishment and maintenance of lines with strong ubiquitous VSR effect. Using the Gal4/UAS binary expression system for establishing spatial control may be a viable alternative (39).

One restriction of our system is that in RNAi experiments, the degree of knock-down on the protein level usually remains unclear. Actually, we usually find a residual level of transcript of around 10 to 20% or more. This might largely reflect the nuclear pre-mRNA, which is protected from RNAi. But it remains likely that some residual protein may remain active. Hence, our experiments may induce not a clear “off-on” situation but rather a “decreased-on” situation. However, we believe that this issue does not interfere with our interpretation, which is based on the phenotypic outcome of the treatment on the cuticle level. Here, we observe a clear break-down and a clear rescue of the segmentation process (i.e., we reached in our experiments the critical level of knock-down and recovery on the protein level).

Of course, it would have been valuable to study the late interactions of the segmentation machinery such as taking out a component during ongoing segmentation. We thought this could be done by dsRNA injection into embryos at different stages. Indeed, we tried several injection time series using *Tc-prd* as target gene. In all batches analyzed, the RNAi knock-down was similar in strength for anterior and posterior segments (i.e., gnathal vs. abdominal segments). The later we injected, the weaker was the phenotype for all body regions alike. Apparently, the build-up of the RNAi response takes longer than the segmentation process, which takes around 15 h at (32 °C) starting at the differentiated blastoderm stage. This would explain why all segments were affected by the same degree of RNAi knock-down. Other experimental approaches would be needed for taking out a component during elongation.

### Segmentation Relies on both Robust and Interruptible Regulatory Feedback Loops.

pRNAi targeting a number of segmentation genes leads to the breakdown of segmentation and loss of all or most trunk segments. This breakdown phenotype was observed after the knock-down of several classes of genes such as the gap-gene orthologs *Tc-hunchback, Tc-Krüppel,* and *Tc-giant* (26–28), the pPRGs *Tc-eve, Tc-odd,* and *Tc-run* (8), the posterior marker *Tc-caudal* (43), and components of the Wnt signaling pathway (42, 45) and torso signaling components (41). In all these cases, the phenotypes were experimentally generated by a continuous knock-down of the respective gene function throughout development by an ongoing RNAi response. Hence, two alternative explanations for the breakdown phenotypes remained possible: On one hand, they could reflect an inherent instability of the system that irreversibly breaks down after the removal of an essential component—the continuous knock-down would not have been required for the loss of most posterior segments. Alternatively, the system could be robust and in principle able to reinitiate. However, the continuous absence of an essential component could still lead to the apparent breakdown phenotype. The results presented here indicate that the gene regulatory system of segmentation actually contains both a robust down-stream component able to reinitiate and a less robust upstream component that can breakdown irreversibly.

Recent elaborations of the clock and speed regulation gradient model contain two genetic feedback loops. First, a positive feedback loop between Wnt signaling and *Tc-cad* expression, which maintains their continued expression in the SAZ (1, 4). The resulting graded activity represents the speed regulation gradient, which influences the velocity of the clock (14). In our experiments, segmentation did not reestablish after the knock-down of two Wnt components although their expression could have resumed after blocking RNAi by the hsVSR. This indicates that the Wnt/cad feedback loop had irreversibly broken down or had not been established in the first place. Torso signaling is required for the establishment of the *T. castaneum* SAZ but ceases to be active in the SAZ after establishment (41). Therefore, even after the rescue of Wnt signaling activity by the hsVSR, the SAZ might fail to reestablish due to the lack of Torso signaling. Interestingly, posterior Wnt signaling is self-activating at least at early stages (18). This autoregulatory loop would theoretically be sufficient to maintain or even establish Wnt activity at the posterior and the design of such a simple positive feedback loop would readily explain an irreversible breakdown. However, a simple one-component self-activating system would be prone to activation at erroneous sites—especially considering the many places of Wnt activity throughout development. Therefore, *Tc-cad* seems to be an essential component of the upstream feedback loop maintaining the SAZ. This additional component would confer a robust localization of the SAZ at the posterior. Indeed, there is evidence that *Tc-cad* and Wnt signaling depend on each other in *T. castaneum* (16, 18, 20). Hence, we predict that in similar hsVSR experiments, *Tc-cad* RNAi would lead to an irreversible knock-down as well. Unfortunately, we were not able to test this hypothesis because strong pRNAi targeting *Tc-cad* resulted in sterility.

The second regulatory feedback loop in the system is the primary PRG gene circuit (8). In the framework of the clock and speed regulation gradient model, this circuit is thought to be the molecular realization of the cell-autonomous clock (20). We found that this feedback loop readily reestablished itself after the knock-down of the components was blocked by hsVSR treatment. This was observed for all three components. Hence, the components of that loop appear to be connected in a way that allows for a reinitiation of the clock whenever all components are functional (i.e., all three pPRGs and the upstream Wnt/cad gradient). The robustness of the PRG loop and/or the downstream events is further supported by the observation that the quite variable early rescue found on the expression level channeled into a quite precise rescue on the cuticle level. Another contribution to the robustness might be provided by the positive autoregulation for *Tc-eve* that we found in our experiments.

It had remained a possibility that an as yet unknown factor expressed in the SAZ was acting upstream of the Wnt/cad feedback loop in order to maintain the SAZ. Our data argue against such a hypothetical factor because the interruption of Wnt signaling led to an irreversible breakdown of the system. If the Wnt/cad system was activated by an upstream factor, one would have expected reinitiation. In line with this hypothesis, the genome-wide iBeetle RNAi screen failed to reveal such a component in *T. castaneum* (47, 48).

Interruption and reestablishment of the PRG loop did apparently not reset the system that “counts” and stops after the correct number of segments have formed. Probably, this task is performed by a system acting in parallel. A good candidate is the gap gene cascade. Indeed, heat shock–induced activation of *Tc-hunchback* was able to reset this “aperiodic clock” such that the gap gene cascade started being expressed again (25).

### Ensuring Specificity and Robustness of the Segmentation.

The different levels of robustness of the two gene regulatory loops to external manipulations reflect the need for on one hand initiating segmentation only once and at one specific location and on the other hand ensuring the need for robustness of the ongoing segmentation toward external perturbations. Indeed, the regulatory interactions initiating segmentation seem to be designed in a way that establishment of a secondary ectopic SAZ is unlikely. At least three different signaling events need to coincide: The asymmetric activity of canonical Wnt signaling at the posterior is the first zygotic readout of maternally driven axis formation and therefore an excellent trigger for locating the SAZ posteriorly (16, 49). *Tc-tor* signaling restricts activation to very early stages because torso signaling is active in the SAZ only early on (41, 50). *Tc-cad* expression is thought to be regulated by the initial Wnt asymmetry—still, it could confer additional robustness to the spatial specificity of SAZ induction (51). Indeed, the initiation system seems to be extremely stable as we are not aware of reports of split posterior trunks in insect embryos. It would be worth testing, whether these three components are indeed sufficient to initiate a SAZ by the joint ectopic activation of these three components making use of the transgenic and genome editing tool kit of *T. castaneum* (52).

In contrast to the initiation, which should happen only once and only at one position, the ongoing segmentation process should be robust against external perturbations. Hence, our finding that the regulatory setup of the clock components allows for reinitiation after external perturbation fits that expectation. Indeed, the irregular stripes reinitiating after rescue in early embryos (Fig. 5*E*) lead to remarkable well-developed segments in the cuticle. Further, when a second SAZ is specified early on either by genetic interference or by classic embryonic manipulations in other insects, a perfectly well-developed mirror image abdomen can develop (16, 53–56). This indicates that indeed, after initiation, the segmentation process is robust and autonomous.

## Materials and Methods

### Strains and Husbandry.

Experimental animals were kept at 32 °C and 40% RH. The transgenic line was generated in the vermillion^white^ strain, which was used for negative controls as well.

### RNAi and Heat Shock Treatment.

pRNAi was performed according to established methods (5, 57). Heat shock conditions were optimized using the number of surviving embryos and the number of rescued segments as criteria. For heat shock, the embryos were put into a beaker and placed into a 48 °C waterbath for 10 min. The embryos were allowed to recover for 2 h at 32 °C before the second heat shock. Thereafter, the embryos were kept at 32 °C until fixation or cuticle preparation. See *SI Appendix* for more details.

### Staining and Microscopy.

Embryo fixation, in situ hybridization, and HCR staining were performed as described previously (58) (HCR protocol shared by Eric Clark and Olivia Tidswell prior to publication) (59, 60). Cuticle autofluorescence was analyzed using either a Zeiss AxioPlan 2 (10× air objective) or a Leica SP5 inverted cLSM (10× air objective). HCR stainings were documented using a Leica SP8 CLSM (20× objectives; 100% glycerol immersion).

### qPCR.

qPCRs were performed using the CFX96 Real-Time PCR System (Bio-Rad Laboratories) with 5× HOT FIREPol^®^ EvaGreen^®^ qPCR Mix Plus (ROX) Master mix (Solis Biodyne). Reference genes were identified using RefFinder (61), and analysis was done in the CFX Manager 3.1 (Bio-Rad Laboratories) and pyQPCR with the delta-delta-Ct method (62).

### Statistical Analysis.

The unpaired, two-sided Mann–Whitney *U* test was used for independent samples. All data points were included. Outliers were determined for the plots using R package *ggplot2*, considering data above 1.5 *IQR of the 75th percentile or below 1.5 *IQR of the 25th percentile as outliers. For the number of stripes in germbands, Pearson's Chi-squared Test for Count Data was used with simulated *P*-values by Monte Carlo simulations (B = 1,000). All graphs and statistical calculations were performed using R (v3.5.2; R Core Team, 2018) and RStudio (v1.1.x; RStudio Team, 2015).

### Access to Materials.

The hsVSR transgenic line is kept in our stock collection and distributed upon request to G.B.

## Supplementary Material

Appendix 01 (PDF)

## Data Availability

All study data are included in the article and/or *SI Appendix*. The transgenic strains are kept in the stock collection of G.B. and will be distributed upon request.
