# Supplementary material for: Temporal control of RNAi reveals both robust and labile feedback loops in the segmentation clock of the red flour beetle
Source: Proc Natl Acad Sci U S A. 2024 Jun 12;121(25):e2318229121. doi: 10.1073/pnas.2318229121 (PMC11194489; doi:10.1073/pnas.2318229121)
Supplement: Supplementary file 1 — Appendix 01 (PDF) [file pnas.2318229121.sapp.pdf]

Supplementary file for

Temporal control of RNAi reveals both robust and labile feedback loops in the segmentation clock of the red flour beetle

Felix Kaufholz[1,2]#, Julia Ulrich[2]#, Muhammad Salim Hakeemi[2], Gregor Bucher[2]*

[1]Göttingen Graduate School for Neurosciences, Biophysics, and Molecular Biosciences (*GGNB*)
[2] University of Göttingen, Johann-Friedrich-Blumenbach Institute, GZMB, Department of Evolutionary Developmental Genetics, Göttingen, Germany

Contents

Supplementary Text 2
- 1) Establishment of CrPV1A as a tool for suppressing RNAi in *T. castaneum* 2
- 2) The VSR CrPV1A 4
- 3) Effect of heat-shock on segmentation gene expression 5
- 4) Detailed methods 7
  - Strains and husbandry 7
  - RNAi and heat-shock treatment 7
  - Staining, and microscopy 7
  - qPCR 8
  - Statistical analysis 8

Supplementary Figures 9
- Fig. S1 Embryonic schemes and replicate of *Tc-paired* experiments 9
- Fig. S2 Repetition of Wnt-signalling experiments 10
- Fig. S3 Repetition of Tc-even-skipped experiment 11
- Fig. S4 Replicates by independent researcher 12
- Fig. S5 Definition of developmental stages based on head expression of *Tc-wg* 13
- Fig. S6 Determining the developmental delay induced by heat-shock 14
- Fig. S7 Quantification of gain of striped pPRG expression after hsVSR treatment 15
- Fig. S8 Rescued Tc-eve embryos and classification 16
- Fig. S9 Measuring the knock-down by qPCR 17

References 18

Supplementary Text

1) Establishment of CrPV1A as a tool for suppressing RNAi in *T. castaneum*

Six VSRs were tested in order to identify a strong Viral Suppressor of RNAi (VSR) in *T. castaneum*. The inhibitors from the *Cricket Paralysis virus* (CrPV1A), the *Flock House virus* (FHV B2), *Drosophila C virus* (DCV1A) and *Nora virus* (VP1) had previously been shown to function as suppressors of the RNAi mechanism in *Drosophila melanogaster* (1–6). Further, the suppressor proteins p25 from the Potato virus X (PVX) and p38 from the Turnip Crinkle virus (TCV) were tested. These plant virus proteins had been known to inhibit the RNAi pathway, but not the microRNA pathway in plants (7–9).

First, we evaluated if any of those VSRs would be able to block RNAi targeting an exogenous gene expressed from a transgenic construct. For this purpose, we crossed a UAS-tGFP responder line (10) (red eye marker) to a driver line that expressed Gal4delta in a thoracic region during late larval, pupal and adult stages (name: *Bauchbinde-Gal4*; black eye marker). The double heterozygous offspring of this mating showed a strong thoracic fluorescence (Fig. A – leftmost columns). In parallel, we generated animals where in addition to UAS-tGFP also the UAS-VSR construct was present (right columns). We performed RNAi targeting tGFP both, in the cross without UAS-VSR (middle column) and with UAS-VSR (right column; see table for overview). The RNAi was predicted to deplete tGFP expression unless the UAS-VSR was able to block RNAi. In this experiment, only CrPV1A blocked RNAi to a degree that tGFP expression remained visible (Fig. A top left panel, red box).

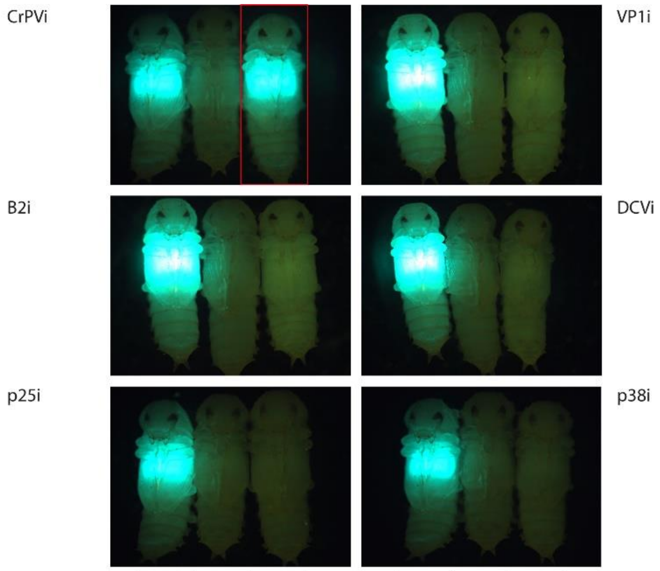

Treatment	1	2	3		1	2	3
BB-Gal4 X UAS-tGFP	X	X	X		X	X	X
BB-Gal4 X UAS-tGFP X UAS-VSR			X				X
tGFP RNAi		X	X			X	X

Figure A Testing six VSRs for inhibition of RNAi. See text for details

In a second experiment, we tested the VSR effect with respect to RNAi targeting an endogenous gene. Larval RNAi against the pigmentation gene *Tc-ebony* was performed. This gene is an N-beta-alanyl dopamine synthetase that is necessary for the synthesis of NBAD sclerotin. RNAi knocking down *Tc-ebony* leads to darkened or black body pigmentation. In this experiment, the UAS-VSR responder lines were crossed to a Gal4 driver, which showed ubiquitous Gal4delta-expression in the epidermis except for early embryonic stages (until appr. 24h at 32°C; we did not check internal organs; name: *Boje-Gal4*). In the presence of an active VSRs a reduction of the portion of black animals and an increase of animals with wildtype color was expected. Again, CrVP1A showed a complete rescue for one insertion (Fig. B, panel D) and partial rescue for another insertion (Fig. B, panel C). The FHV B2 VSR was active to a lesser degree (Fig. B panels E,F - two different insertions led to an increase of wt coloured animals).

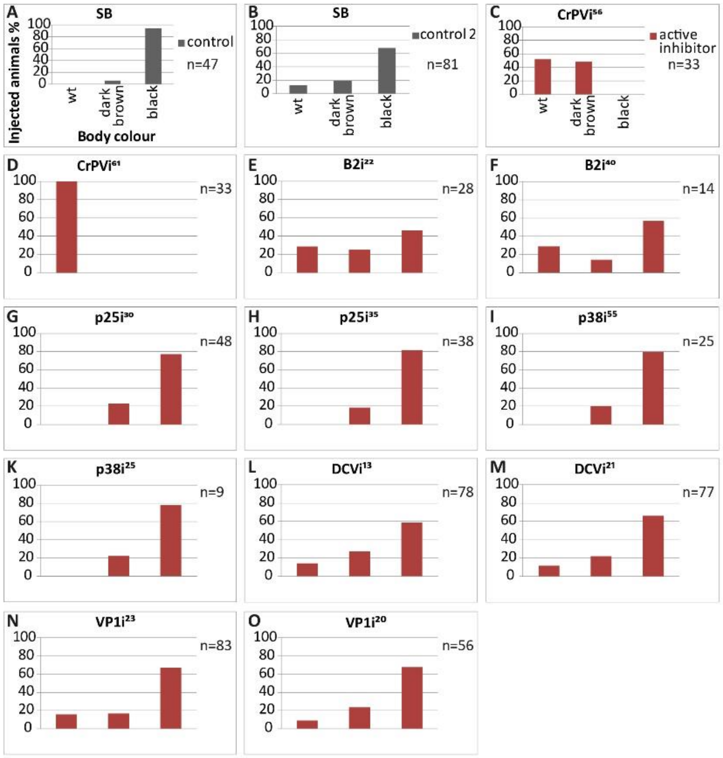

Figure B Testing six VSRs for inhibition of RNAi
See text for details

Taken together, these tests revealed CrPV1A as an efficient suppressor of RNAi in *T. castaneum* transgenically expressed. Based on these results, we chose CrPV1A as tool for temporal control of RNAi in *T. castaneum*.

2) The VSR CrPV1A

Cricket Paralysis virus (CrPV) was initially identified and isolated from field crickets, *Teleogryllus oceanicus* and *Teleogryllus commodus,* and it is a highly potent virus of many species in the laboratory (4, 11, 11). CrPV is closely related to *Drosophila C virus* and likewise belongs to the positive-strand *Dicistroviridae* family. In contrast to DCV, CrPV leads to mortality upon infection of crickets and flies (4, 12). This high pathogenesis of CrPV is partially based on its efficient RNAi suppressor protein, CrPV1A. It has been shown that adding CrPV1A to the Sindbis virus, which does not naturally encode an endogenous RNAi suppressor, resulted in increased virus production and fly lethality upon infection (4). The mode of action of CrPV1A relies on its interaction with the endonuclease Ago-2, a component of the RISC complex. This interaction blocks Ago-2 cleavage activity, resulting in inhibited RISC-mediated mRNA degradation and therewith RNAi disruption. Nevertheless, the suppressor protein CrPV1A did not interfere with the miRNA pathway or alter the physiology and development of the animals when expressed in flies (4). In order to check for lethal effects of VSR expression in *T. castaneum,* we heat-shocked animals of the *vw* strain wildtype and of the hsVSR-line at different stages of development (adult, pupae and larvae). The survival rate was reduced significantly in heat-shock-VSR line animals compared to wildtype (Figure C) indicating adverse effects.

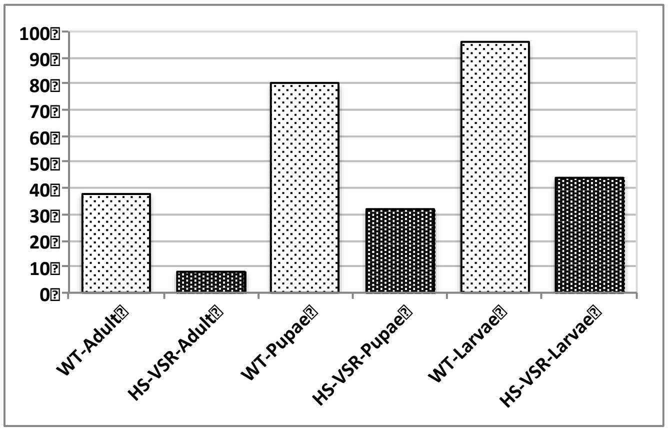

Figure C Survival rate after heat-shock activation of CrPV1A Light grey columns indicate the survival rate of wildtype animals after heat-shock whereas dark filled columns indicate the survival rate of hsVSR animals after heatshock treatment. After heat-shock activation of viral suppressor of RNAi, the survival rate was reduced significantly in heat-shock-VSR line animals compared to wildtype animals indicating an adverse effect of CrPV1A expression.

3) Effect of heat-shock on segmentation gene expression

To test for the effect of heat-shock on patterning gene expression we performed the procedure given in Fig. 1F for wildtype animals (*SB* strain) with or without heat-shock and stained them for *Tc-wg*, *Tc-eve* and *Tc-cad*. Then, embryos were scored for wildype and aberrant patterns. *Tc-eve* has a very dynamic pattern such that minor modifications are difficult to detect. Therefore, we scored for embryos that showed 2 or more stripes (reflecting the expected pattern for ongoing segmentation) versus animals that had clearly aberrant patterns or only one posterior domain (reflecting either aberrant pattern in elongating embryos or a regular pattern observed at the end of segmentation). We found no difference between heat-shocked or non-heat-shocked. Similarly, all embryos of both treatments had the expected single posterior domain. For *Tc-wingless*, we found that 50% of the heat-shocked embryos had localized defects in some abdominal segments (Fig. D) while in non-heat-shocked controls this was not observed.

These observations would be in line with a temporally restricted effect of heat-shock on segmentation. The genes acting early in the hierarchy (*Tc-cad, Tc-eve*) had probably already recovered from potential earlier defects while the observed spatially restricted *Tc-wg* defects probably reflected patterning problems induced during the temporally limited heat-shock. This confirms our observation from cuticle analysis that a heat-shock leads to unspecific defects in a subset of embryos.

	**Non heat-shocked**		**Heat-shocked**	
Tc-eve	2 or more stripes	No expression or only 1 domain	2 or more stripes	No expression or only 1 domain
	15	13 (46 %)	21	21 (50%)
Tc-cad	Wildtype	aberrant	Wildtype	aberrant
	> 20	0	> 20	0
Tc-wg	Wildtype	aberrant	Wildtype	aberrant
	21	0	9	9 (50%)

Figure D Developmental defects induced by heat-shock

A) Embryo from the non-heat-shock control showing the typical pattern of segmental *Tc-wg* staining in addition to the domain in the growth zone. B-D) Embryos from the heat-shocked sample showing deviations of segment formation in the abdomen. Regular stripes posterior to the defect domain indicate that segmentation resumed after some time.

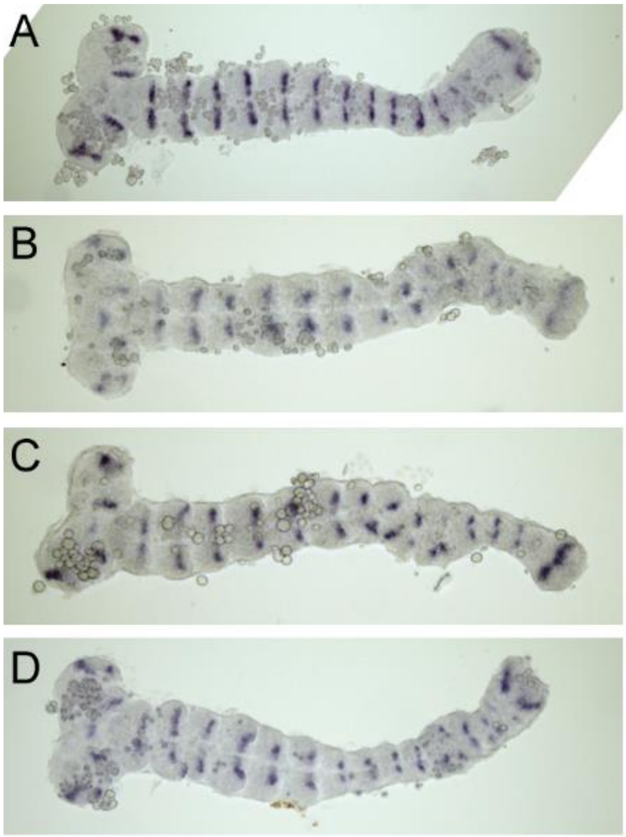

4) Detailed methods

Strains and husbandry

Tribolium castaneum (HERBST) beetles were reared using standard conditions and methods (52). During experiments, beetles (embryos/larvae) were kept at 32°C and 40% RH while general stock keeping was done at 28°C and 40% RH. RNAi inhibition experiments were performed in the transgenic line containing the RNAi inhibitor CrPV1A from the Cricket Paralysis Virus under the control of the endogenous *Tribolium* heat shock promoter (40) and the 3xP3DsRed (eye marker) integrated into the genome using piggyback vector (57) in *Tribolium* line vermillion^{white}. Non-transgenic vermillion^{white} beetles were used for negative controls.

RNAi and heat-shock treatment

Parental RNAi was performed according to established methods (5, 58). Templates were prepared by PCR with T7 overhanging-primers from plasmid templates containing varying lengths of coding and/or regulatory mRNA sequence (*Tc-even-skipped* ~1400 bp, *Tc-odd-skipped* ~380 bp, *Tc-paired* ~540 bp, *Tc-arrow* ~1800 bp, *Tc-Wnt8/D* ~500 bp, *Tc-wntless* ~600 bp). DsRNA was produced using MEGAscript T7 Transcription Kit (Life Technologies). The concentration of injected dsRNA for parental RNAi was 1000 ng/µl (*Tc-even-skipped*), 500 ng/µl (*Tc-odd-skipped, Tc-paired*), or 100 ng/µl (*Tc-arrow, Tc-Wnt8/D, Tc-wntless*). Add NCBI accession number? *Tc-eve*: NM_001039449.1; *Tc-odd*: XM_008198532.2; *Tc-run*: XM_964184.3; *Tc-wg*: NM_001114350

Heat-shock conditions for *T. castaneum* had been extensively optimized in a previous work (40). Starting from these values, we optimized the conditions using the number of surviving embryos and the number of rescued segments as criterion. Embryos from dsRNA injected animals of either the hsVSR or wild type control (*vermillion^{white}*) were collected and kept without flour at 32°C in small plastic fly culturing vials before the treatment. For the heat-shock, the eggs were transferred to a small (40ml) glass beaker with a flat bottom making sure that all embryos had direct contact with the bottom. Then, the beaker was put into a pre-heated 48°C waterbath. The beaker was covered with perforated aluminum foil. The bottom of the glass beaker was kept submerged for 10 min. To ensure a controlled and quick termination of the heatshock, the beaker was put into a room temperature waterbath. After transferring the embryos back into the plastic vials, they were allowed to recover for two hours at 32°C, until they were heatshocked a second time for 10 min at 48°C, following the same procedure. Based on (40), the VSR mRNA is predicted to be expressed for about 5 hours by that treatment. In the absence of an VSR antibody we were not able to measure the protein dynamics. Thereafter, the embryos were kept at 32°C until fixation or cuticle preparation. We note that the results were sensitive even to minor changes of the procedure and each step needed to be optimized carefully.

Staining, and microscopy

Embryo fixation and in-situ hybridisation were performed as described previously (59). Digoxigenin (DIG)-labeled riboprobes targeting *Tc-wg* (DIG RNA Labeling Kit, Roche), was detected by anti-DIG-AP antibodies (Roche) and visualized by NBT/BCIP staining. HCR staining

was performed as published with small modification (kindly provided by Eric Clark and Olivia Tidswell prior publication) (60, 61). HCR probes for *Tc-eve* were purchased from Molecular Technologies while HCR probes for all other genes were purchased from Molecular Instruments. Binding sequences are available from vendors at request due to intellectual properties restrictions. Cuticles of L1 larvae were analyzed and documented using either a Zeiss AxioPlan 2 (10x air objective) with ImagePro 6 or a Leica SP5 inverted cLSM (10x air objective) with Leica LAS-X software, utilizing the cuticle's autofluorescence. HCR stainings were documented using a Leica SP8 confocal laser-scanning microscope (20x objectives with 100% glycerol as immersion medium) and the Leica LAS-X software (v 3.5.2). In-situ hybridization was documented using Zeiss AxioPlan 2 or Zeiss AxioScope.

qPCR

RNA from whole embryos was extracted using the Quick-RNA Tissue/Insect Kit (Zymo Research) with DNase on-column digest (DNaseI Set, Zymo Research). cDNA was synthesized using the MAXIMA First Strand cDNA Synthesis Kit for RT-qPCR (Thermo Fisher Scientific) according to manufacturer’s instructions. qPCRs were performed using the CFX96 Real-Time PCR System (Bio-Rad Laboratories) with 5x HOT FIREPol® EvaGreen® qPCR Mix Plus (ROX) Master mix (Solis Biodyne). Reference genes were identified using RefFinder (62). qPCR data analysis was done in the CFX Manager 3.1 (Bio-Rad Laboratories) and pyQPCR with the delta-delta-Ct method (63).

Statistical analysis

Comparisons of abdominal segment numbers in cuticles and comparisons of number of expression stripes in germbands were tested using unpaired, two-sided Mann–Whitney U tests for independent samples. All measured data points were included in the calculations and were not checked for outliers beforehand. Outliers were determined for the plots using the R package *ggplot2*, considering data above 1.5 *IQR of the 75th percentile or below 1.5 *IQR of the 25th percentile as outliers, which are indicated in in the respective plots in red. Comparisons of the number of stripes in germbands were done using the Pearson's Chi-squared Test for Count Data with simulated p-values by Monte Carlo simulations (B=1000). All graphs and statistical calculations were performed using R (v3.5.2; R Core Team, 2018) and RStudio (v1.1.x; RStudio Team, 2015) with the following packages: dplyr, ggplot2, ggpubr, ggsigni, patchwork, readxl, reshape2.

Supplementary Figures

Fig. S1 Embryonic schemes and replicate of *Tc-paired* experiments

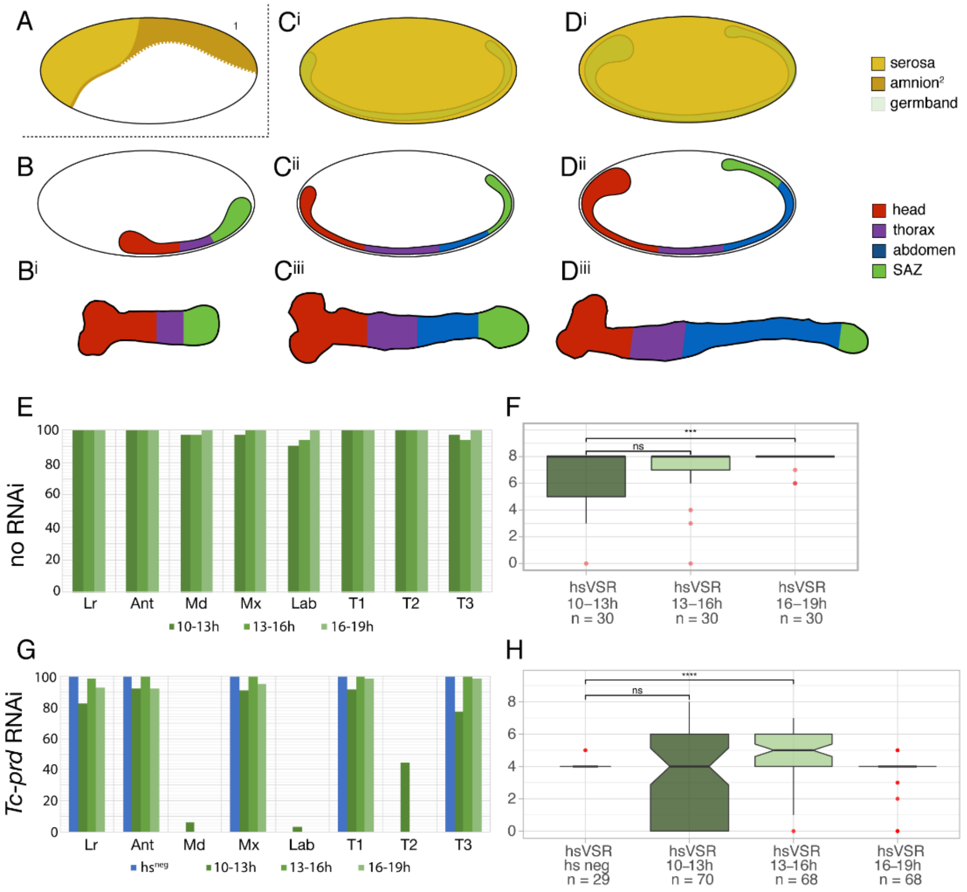

Fig. S1 Scheme of early development and repetition of control experiments

A) In *T. castaneum*, the embryonic anlagen are located at a ventral posterior position in the blastoderm. The extraembryonic tissues serosa (light yellow) and amnion (dark yellow) are depicted. B) The early germ band can be separated in head and thoracic segments (red and purple), which are specified during the blastoderm stage and the SAZ (green). C,D) During elongation, abdominal segments are added in a sequential way in the SAZ. The depicted stages correspond approximately to the stages in which heat-shocks were performed. E) Heat-shocks alone did not much affect anterior segments. F) Importantly, the earlier heat-shocks did affect the number of abdominal segments indicating that this treatment disturbs segmentation to some degree. G and H) Repetition of the Tc-prd experiment (see text for details). In this repetition, the negative influence of the heat-shock is more pronounced than in the experiment shown in Fig. 2.

Fig. S2 Repetition of Wnt-signalling experiments

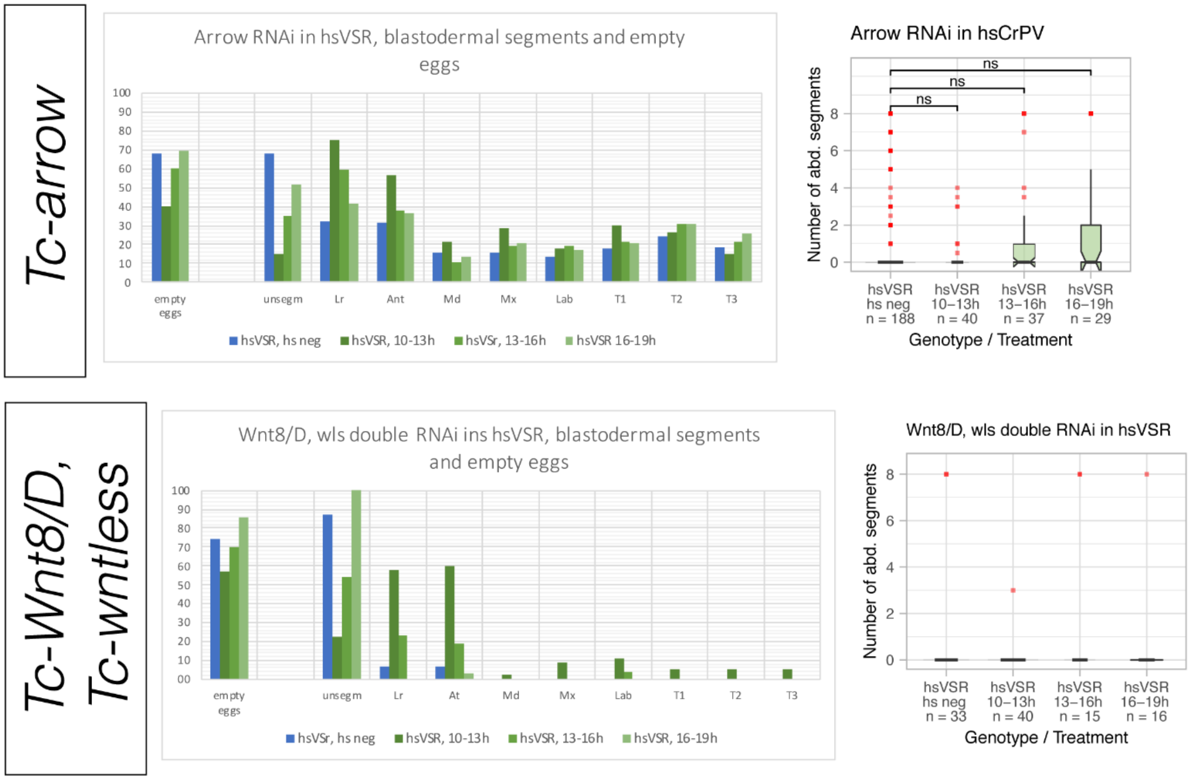

Fig. S3 Repetition of Tc-even-skipped experiment

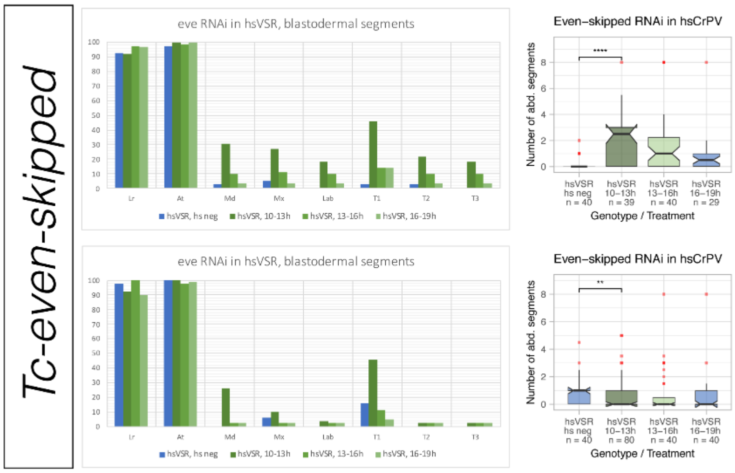

Fig. S4 Replicates by independent researcher

Controls:

Tc-paired

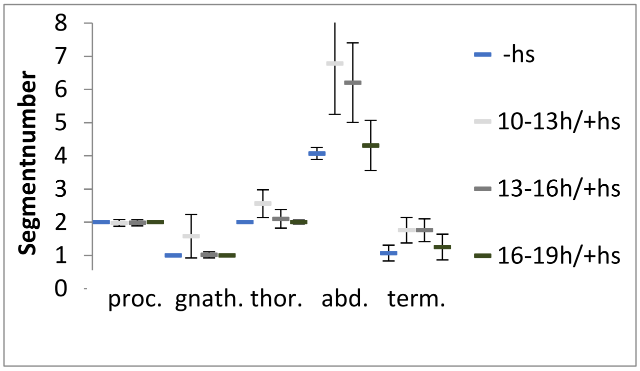

wildtype

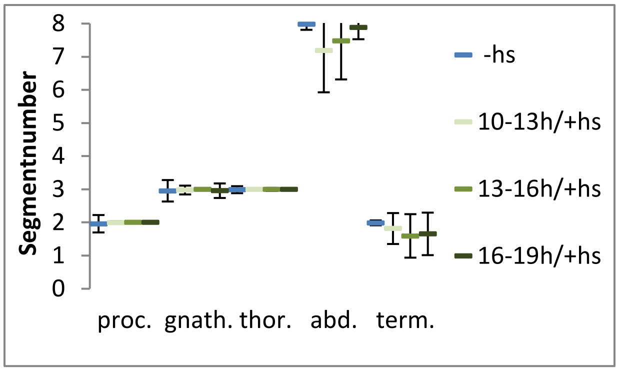

Tc-odd

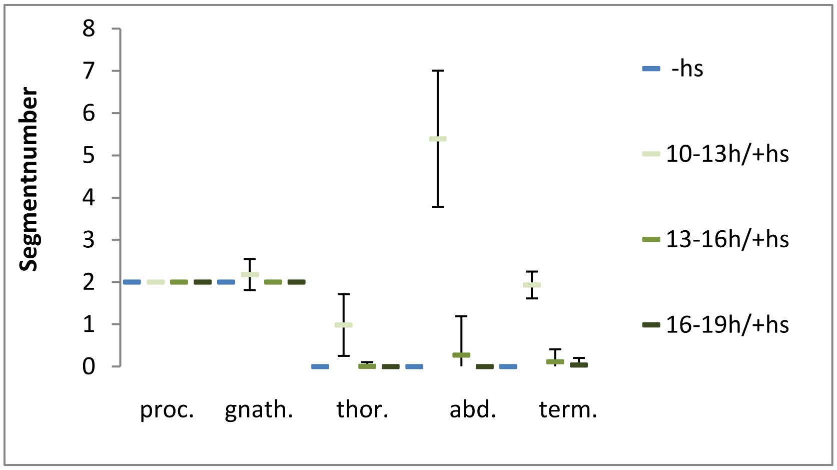

Tc-runt

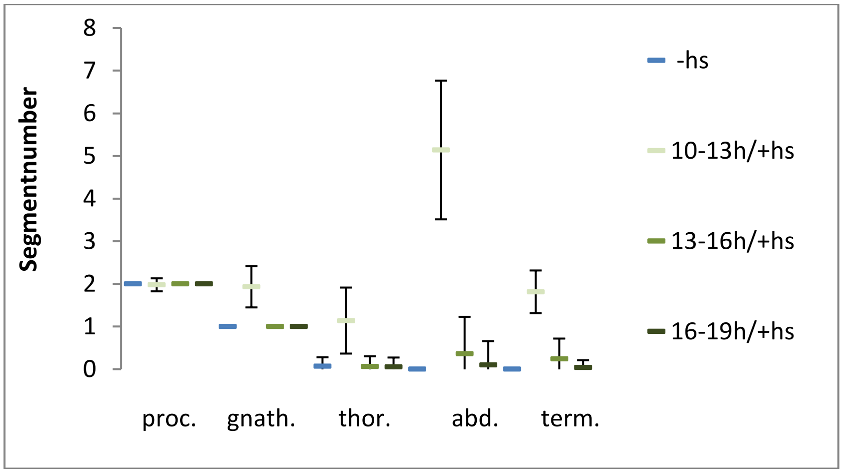

Fig. S5 Definition of developmental stages based on head expression of *Tc-wg*

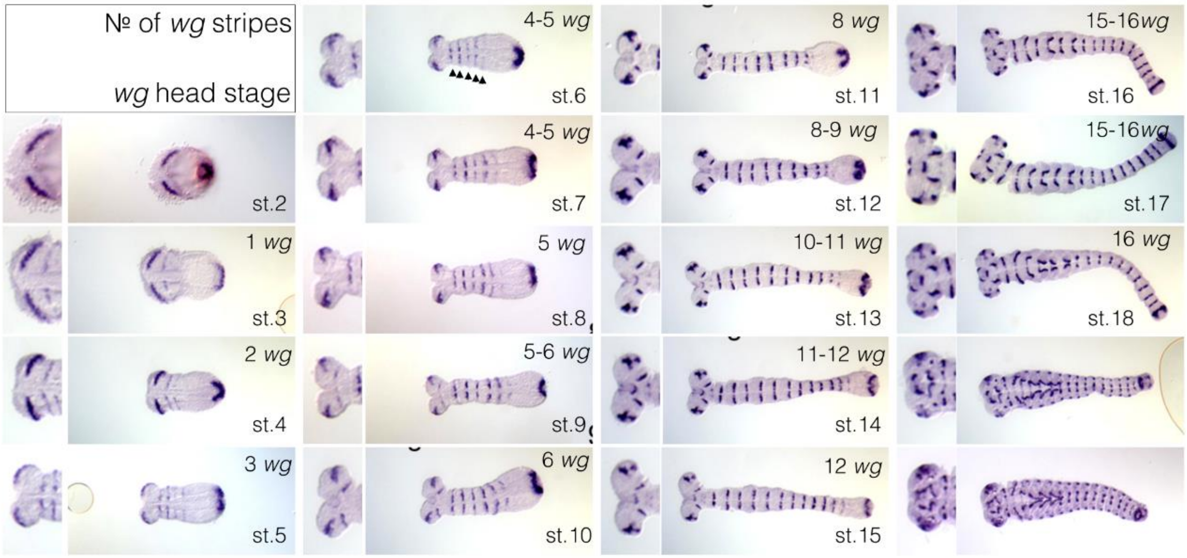

Fig. S6 Determining the developmental delay induced by heat-shock

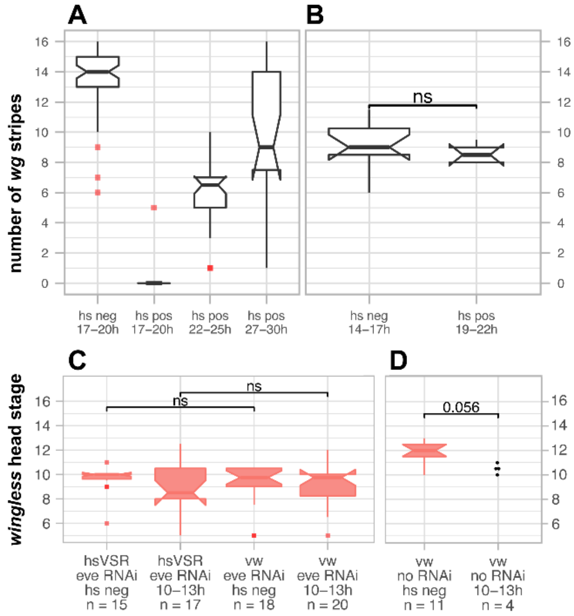

A) To determine the delay, we first compared development between wt and heat-shocked embryos using the number of abdominal *Tc-wg* stripes. For late stages, this revealed a lag of more than 10 hours. B) For embryos during ongoing segmentation, fixation with a 5h difference resulted in comparable stages. C) We used that timing to stage heat-shocked and non-heat-shocked wt and *Tc-eve*-RNAi embryos. Because these RNAi embryos miss abdominal segments, we used the dynamic *Tc-wg* head expression to stage the embryos (see Fig. S5). D) The shift of developmental timing was confirmed by counting Tc-wg stripes in embryos without RNAi.

Fig. S7 Quantification of gain of striped pPRG expression after hsVSR treatment

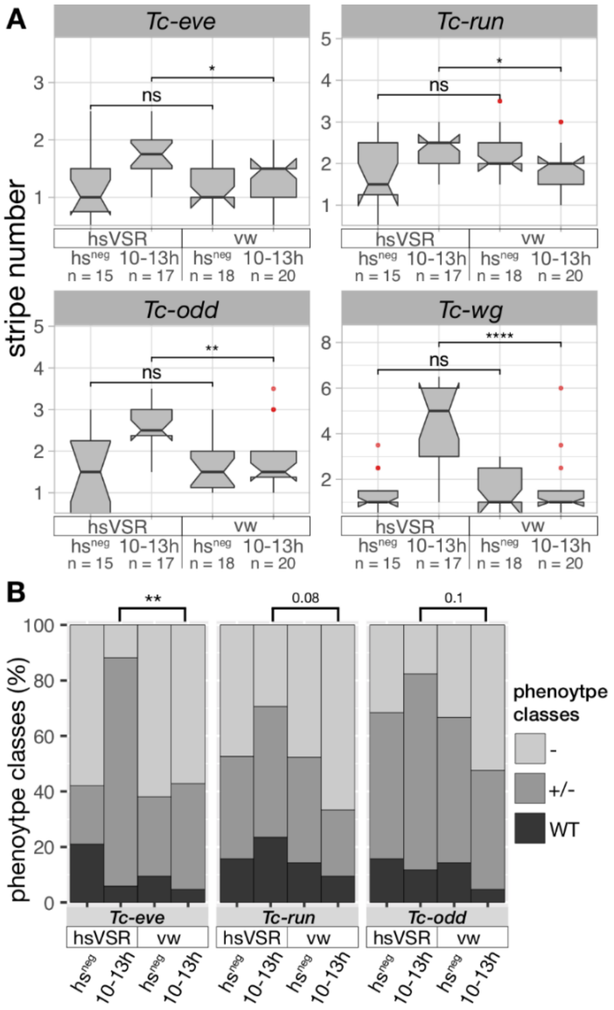

A) The number of pair rule gene and *Tc-wg* stripes increased significantly when comparing the heat-shocked batches from the hsVSR line and wildtype. B) In an alternative analysis, the resulting embryos were assigned to three classes: no stripes (-), intermediate (+/-) and close to wildtype (WT). The p-value for Tc-eve reached significance levels while for the other genes the p-value was low but not significant.

Fig. S8 Rescued Tc-eve embryos and classification

See text for details

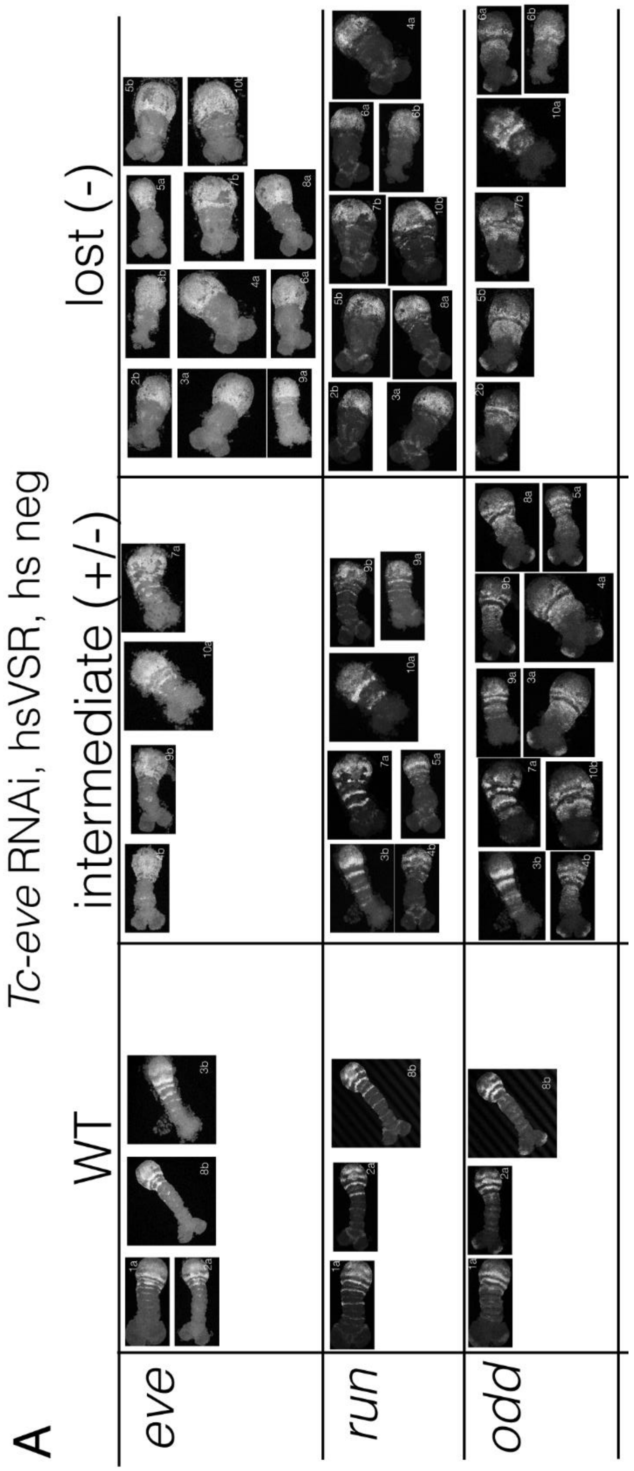

Fig. S9 Measuring the knock-down by qPCR

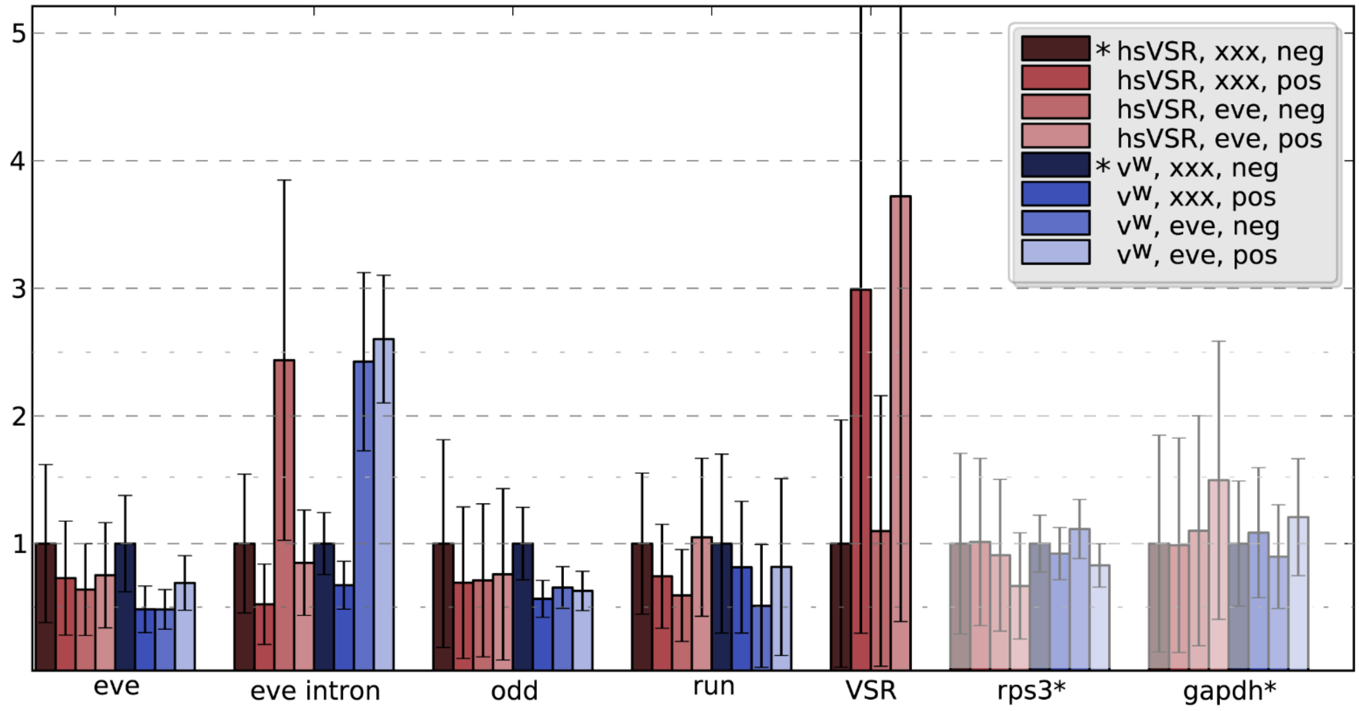

qPCR was performed in the hsVSR line (reddish colors) and vw wildtype (blue colors). Rps3 and gapdh were used for normalization. As expected, heat-shock treatment increased the amount of VSR transcript (VSR – compare bars with heatshock ("pos") with those without heatshock ("neg"). Surprisingly, Tc-eve expression was not much reduced after Tc-eve RNAi. However, nuclear transcripts did increase dramatically in eve RNAi embryos that were not rescued (eve intron, intermediate red bar). Tc-odd expression was not much altered in line with ongoing expression in the knock-down embryos. Tc-runt may be downregulated in Tc-eve RNAi (middle bar) but the expression was rescued in Tc-eve RNAi combined with hsVSR rescue (light red bar).

References

1. R. Aliyari, *et al.*, Mechanism of induction and suppression of antiviral immunity directed by virus-derived small RNAs in Drosophila. *Cell Host Microbe* **4**, 387–397 (2008).

2. J. A. Chao, *et al.*, Dual modes of RNA-silencing suppression by Flock House virus protein B2. *Nat Struct Mol Biol* **12**, 952–957 (2005).

3. H. Li, W. X. Li, S. W. Ding, Induction and suppression of RNA silencing by an animal virus. *Science* **296**, 1319–1321 (2002).

4. A. Nayak, *et al.*, Cricket paralysis virus antagonizes Argonaute 2 to modulate antiviral defense in Drosophila. *Nat Struct Mol Biol* **17**, 547–554 (2010).

5. J. T. van Mierlo, *et al.*, Convergent evolution of argonaute-2 slicer antagonism in two distinct insect RNA viruses. *PLoS Pathog* **8**, e1002872 (2012).

6. R. P. van Rij, *et al.*, The RNA silencing endonuclease Argonaute 2 mediates specific antiviral immunity in Drosophila melanogaster. *Genes Dev* **20**, 2985–2995 (2006).

7. E. H. Bayne, D. V. Rakitina, S. Y. Morozov, D. C. Baulcombe, Cell-to-cell movement of potato potexvirus X is dependent on suppression of RNA silencing. *Plant J* **44**, 471–482 (2005).

8. H. Jin, J.-K. Zhu, A viral suppressor protein inhibits host RNA silencing by hooking up with Argonautes. *Genes Dev* **24**, 853–856 (2010).

9. O. Voinnet, C. Lederer, D. C. Baulcombe, A viral movement protein prevents spread of the gene silencing signal in Nicotiana benthamiana. *Cell* **103**, 157–167 (2000).

10. J. B. Schinko, *et al.*, Functionality of the GAL4/UAS system in *T. castaneum* requires the use of endogenous core promoters. *BMC Dev Biol* **10**, 53 (2010).

11. N. Plus, G. Croizier, C. Reinganum, P. D. Scott, Cricket paralysis virus and drosophila C virus: serological analysis and comparison of capsid polypeptides and host range. *J Invertebr Pathol* **31**, 296–302 (1978).

12. T. Manousis, N. F. Moore, Cricket Paralysis Virus, a Potential Control Agent for the Olive Fruit Fly, Dacus oleae Gmel. *Appl Environ Microbiol* **53**, 142–148 (1987).
